# VOZ1 and VOZ2 transcription factors regulate arsenic tolerance and distribution in rice and Arabidopsis

**DOI:** 10.3389/fpls.2023.1209860

**Published:** 2023-09-20

**Authors:** Ying Wen, Chayanee Chairattanawat, Kieu Thi Xuan Vo, Jiayou Liu, Jie Zhang, Ting Pan, Do-Young Kim, Enrico Martinoia, Chun-Yan Zhong, Mao-Hui Wang, Jong-Seong Jeon, Won-Yong Song

**Affiliations:** ^1^ Department of Horticulture, Foshan University, Foshan, Guangdong, China; ^2^ Department of Integrative Bioscience and Biotechnology, Pohang University of Science and Technology, Pohang, Republic of Korea; ^3^ Graduate School of Green-Bio Science and Crop Biotech Institute, Kyung Hee University, Yongin, Republic of Korea; ^4^ Advanced Bio-convergence Center, Pohang Technopark, Pohang, Republic of Korea; ^5^ Institute of Plant Biology, University Zurich, Zurich, Switzerland; ^6^ Zhaoqing Institute of Agricultural Sciences, Zhaoqing, China

**Keywords:** arsenic, arsenic tolerance rice genes, phloem, rice, seed, zinc finger-type transcription factor

## Abstract

Rice is the major source of arsenic (As) intake in humans, as this staple crop readily accumulates As in the grain. Identifying the genes and molecular mechanisms underlying As accumulation and tolerance is a crucial step toward developing rice with reduced As levels. We identified 25 rice genes that improve As tolerance in yeast cells by expressing a complementary DNA (cDNA) library generated from As-treated rice roots. Among them, a zinc finger–type transcription factor VASCULAR PLANT ONE- ZINC FINGER 1 (OsVOZ1) (OsVOZ1) conferred the most pronounced As tolerance. OsVOZ1 inhibits As accumulation in yeast via activation of As efflux transporter Acr3p by post-transcriptional modification in yeast. The Arabidopsis *voz1 voz2* double-knockout mutant exhibited As hypersensitivity, altered As concentrations in various tissues, and reduced As transport activity via the phloem. Arabidopsis and rice *VOZs* were highly expressed in phloem cells in various tissues, which are critical for As distribution in plant tissues. The double-knockdown and single-knockout plants of *OsVOZ1* and *OsVOZ2* reduced As accumulation in their seeds. These findings suggest that rice and Arabidopsis VOZs regulate the translocation of As into tissues by regulating the phloem loading of this element.

## 1 Introduction

Arsenic (As) causes various diseases in humans, such as cancer and skin lesions, and is classified as a non-threshold class 1 carcinogen (Mandal and Suzuki, 2002; Smith et al., 2002). Rice (*Oryza sativa*), a staple food consumed by the population of many nations throughout the world, is a major source of As intake in humans (Schoof et al., 1999; Meharg, 2004) because it accumulates As in the grains more efficiently than other crops (Williams et al., 2007; Ma et al., 2008; Zhao et al., 2010). It is therefore crucial to search or develop rice varieties with reduced As levels to reduce potential human health risk caused by As toxicity. To develop such rice varieties, it is important to identify the genes and mechanisms responsible for the uptake, translocation, and accumulation of As in grains.

As enters the rice plant via the same pathways used for the uptake of nutrients such as inorganic phosphate (Pi) and silicon (Si). Under aerobic soil conditions, arsenate [As(V)] enters primarily into the roots of plants through high-affinity plasma membrane-localized phosphate transporters, known as Pht1 transporters (Zhao et al., 2010). OsPht1;8 and OsPht1;1 have high affinities for both Pi and As(V); rice plants overexpressing these proteins exhibit markedly increased As(V) uptake (Wu et al., 2011; Kamiya et al., 2013).

The major portion of As(V) taken up by Pht1 transporters is rapidly reduced to arsenite [As(III)] by As reductase in the cytosol of the root and translocated into the shoot via the xylem. ATQ1/HAC1 (Arsenic Tolerance QTL 1/High Arsenic Content1), an As reductase identified in *Arabidopsis thaliana*, promotes plant tolerance to As(V) by reducing As(V) to As(III) (Chao et al., 2014; Sanchez-Bermejo et al., 2014). Loss-of-function mutants of ATQ1/HAC1, which exhibit reduced As(V) reductase activity, accumulate higher levels of As(V) and show reduced As(III) extrusion from the root compared with the wild type (WT), because plants extrude only As(III) efficiently, but not As (V).

The Pi transport pathway is not a major route for As entry in rice under normal cultivation conditions, as As(V) represents only a minor As species under anaerobic conditions prevailing in paddy soil flooded with water. Under such anaerobic conditions, As(III) is the dominant As species, which enters the root and moves to the shoot via Si/As(III) transporters (Zhao et al., 2010). Distally localized *Oryza sativa* Low silicon1 (OsLsi1) is a major As(III) channel through which As(III) enters exodermal and endodermal cells in the root. As(III) is pumped toward the stele by proximally localized OsLsi2 (Ma et al., 2008). The loss of function of *OsLsi1* strongly reduces As(III) accumulation in roots and shoots, and the loss of function of *OsLsi2* reduces As translocation to the shoot (Ma et al., 2008). The overexpression of the As(III)-permeable aquaporins OsNip1;1 and OsNip3;3 reduces root-to-shoot As translocation and accumulation in the grain due to reduced xylem loading of As (Sun et al., 2018).

The phloem is the major route for As distribution into seeds (Carey et al., 2011; Mendoza–Cozatl et al., 2011; Li et al., 2016). Therefore, loading and unloading of As to the phloem largely determines As levels in grains. Phloem transport is thought to account for 90% of the As(III) unloaded into rice grains (Carey et al., 2010). *Arabidopsis thaliana* inositol transporters (AtINTs) are involved in the phloem loading pathway of As (Duan et al., 2016). Both *atint2* and *atint4* mutants exhibit reduced As contents in siliques and seeds. Nodes are well-organized vascular systems that connect the roots, leaves, tillers, and panicles of gramineous plants, including rice, and distribute minerals into grains (Moore et al., 2014). As levels are higher in nodes than in other tissues. In particular, phloem cells of diffuse vascular bundles (DVBs) in the node accumulate high levels of As. OsABCC1, a putative vacuolar arsenite-phytochelatins [As(III)–PCs] transporter localized to the phloem companion cells of vascular bundles in the node, inhibits the translocation of As into grains (Song et al., 2014). Consistently, the *osabcc1* knockout mutants accumulate higher levels of As in their grains but lower levels of As in their nodes than the WT. OsLsi2, which is highly expressed in parenchyma cells bridging the border between enlarged vascular bundles (EVBs) and DVBs in nodes, mediates the intervascular transfer of As(III) (Chen et al., 2015). The *lsi2* knockout mutants contain more As than the WT in the flag leaf but less in the grain. Therefore, identifying genes that participate in the phloem transport pathway of As should facilitate the generation of plants with reduced As contents in the grains.

Transcription factors play crucial roles in regulating the expression of As transporters involved in the uptake, efflux, and compartmentalization of As (Castrillo et al., 2013; Wang et al., 2014; Wang et al., 2017). However, only a few transcription factors involved in this process have been identified in plants to date. The transcription factor *Arabidopsis thaliana* WRKY6 (AtWRKY6), which is induced by As(V), represses the expression of the Pi transporter gene *AtPHT1;1*, thereby repressing As(V) and Pi uptake in Arabidopsis (Castrillo et al., 2013). Thus, Arabidopsis plants overexpressing *AtWRKY6* exhibit enhanced As(V) tolerance. AtWRKY45, a transcription factor that upregulates the expression of *AtPHT1;1*, enhances As and Pi uptake (Wang et al., 2014); thus, *WRKY45*-overexpressing plants are much more sensitive to As(V) compared with WT plants. As(III), the major As species in flooded paddy soils, induces the expression of the R2R3 MYB transcription factor OsARM1, which upregulates the As-associated transporter genes *OsLsi1*, *OsLsi2*, and *OsLsi6* in rice roots and shoots (Wang et al., 2017). *Osarm1* knockout plants show improved As(III) tolerance and increased root-to-shoot As translocation, whereas plants overexpressing *OsARM1* exhibit increased sensitivity to As(III) and reduced As translocation to shoots. These findings suggest that transcription factors that regulate the expression of As(V)/As(III) transporters strongly affect the uptake and root-to-shoot translocation of As, as well as As tolerance.

Much is known about the genes and mechanisms for As detoxification and transport in *Saccharomyces cerevisiae* compared with other organisms. Yeast cells reorganize proteostasis pathways to minimize As uptake, enhance As efflux, and reduce proteotoxicity caused by misfolded proteins. Proteins involved in As efflux transport, autophagy, proteasome, molecular chaperone, and sulfur metabolism are increased by the As treatment, whereas ribosomal proteins, ribosome biosynthesis, and amino acid biosynthesis and glucose transporters are decreased (Guerra-Moreno et al., 2015; Jochem et al., 2019). As(III) is taken up by aquaporin (FPS1) and hexose transporters (HXTs) (Wysocki et al., 2001; Liu et al., 2004) and detoxified by As efflux via Arsenical Resistance Protein (ACR3) and by vacuolar sequestration via Yeast Cadmium Factor (YCF1) (Li et al., 1996; Wysocki et al., 1997; Ghosh et al., 1999). HXTs, transporters responsible for up to 80% of As(III) uptake at the plasma membrane, are degraded by the ubiquitin-vacuole pathway to decrease As(III) uptake in yeast cells. The degradation of HXTs requires the E2 ubiquitin ligase Ubc4, the E3 ubiquitin ligase Rsp5 and K63-linked ubiquitin chains, and expression of a degradation-resistant 9K-to-R mutant form of HXT2 restores As sensitivity to the *hxt1-7* mutant (Jochem et al., 2019). Hog1, the yeast stress-activated protein kinase that is phosphorylated by stresses including As(III) exposure, enhances As (III) resistance through the regulation of As uptake and efflux mediated by Acr3 and FPS1 (Thorsen et al., 2006). HOG1 increases As efflux through the upregulation of the ACR2 and ACR3 transcript levels, mediated by phosphorylation of the YAP8 transcription factor (Guerra-Moreno et al., 2019). HOG1 also prevents As(III) influx via phosphorylation of T231 at the N-terminal domain of FPS1, whereas the mitogen-activated kinase SLT2 promotes As(III) efflux through phosphorylation of S537 at the C-terminus (Ahmadpour et al., 2016).

Many proteins important for As tolerance and transport have been identified in plants. These proteins are restricted to a few transporters (aquaporins, Pi transporters, and C-type ABC transporters) and enzyme (As reductase) families, but their regulations are poorly understood. The aim of this study was to identify the gene pool and mechanisms involved in As movement in rice. To reach this goal, we screened for novel As tolerance genes in budding yeast by expressing a rice cDNA library generated from As-treated rice roots and selected colonies that grew in As-containing medium. Genes regulating transcription (transcription factors) and post-transcriptional modification (polyubiquitin, 26S proteasome complex subunit, and autophagy) were identified with high portion in this screening. Here, we selected two transcription factors—VOZ1 (Vascular Plant One-Zinc finger1) and VOZ2—among the genes identified to elucidate their role in As tolerance and As translocation because they conferred the strongest tolerance to As in yeast cells and were specifically expressed in vascular tissues in rice and Arabidopsis. The knockout and knockdown of both *VOZ1* and *VOZ2* reduced As concentration in Arabidopsis and rice seeds. This work provides new information about regulatory pathways used by plants to tolerate As and to accumulate As in their seeds and suggests a strategy for altering this pathway to reduce As accumulation in rice grains.

## 2 Materials and methods

### 2.1 Screening for novel arsenic tolerance genes in rice

To screen novel As tolerance genes in rice, total RNA was extracted from the roots of 4-week-old rice plants treated with 10 μM As(III) for 1 to 24 h. The RNA was used to synthesize cDNA with a cDNA library construction kit (Takara), which was inserted into the *Eco*RI/*Xho*I sites of the pYES2 vector, allowing for galactose-inducible gene expression. The cDNA library was introduced into yeast strain SM15 (*MATa*, *ura3*, *leu2*, *his3*, *trp3*, *lys2*, *suc2*, *ycf1::hisG*, *yhl035c::Leu3*, *yll015w::Kan-MX6*, *yll048c:: TRP1-MX6*, and *TaPCS1::cup1–1*; Mendoza-Cózatl et al., 2010), and transformants surviving on synthetic galactose minus uracil medium (SG^ura-^) supplemented with 70 to 100 μM As(III) were selected. False-positive clones that survived on synthetic dextrose minus uracil medium (SD^ura−^) containing 100 μM As(III) were eliminated. The plasmids were rescued from the selected transformants and sequenced at the second screening step (**Supplementary Figure 1**, top right).

### 2.2 Expression and characterization of As tolerance genes in yeast

The selected candidate genes, including a gene encoding an nicotinamide adenine dinucleotide (NAD)-dependent epimerase/dehydratase family protein (epimerase, LOC_Os03g17000), *OsVOZ1* (LOC_Os01g54930), *OsVOZ2* (LOC_Os05g43950), and *OsBIM2* (LOC_Os09g29930), and genes encoding polyubiquitin (RUBQ2, LOC_Os02g06640), H/ACA ribonucleoprotein complex subunit 4 (H/ACA snoRNP complex subunit 4, LOC_Os07g44190), and autophagy-related protein 8A (autophagy, LOC_Os07g32800), were transformed into different As-sensitive yeast strains, such as SM15 expressing TaPCS1, RW105 (*acr3 ycf1*; Maciaszczyk-Dziubinska et al., 2010), and *ubi4* (**Supplementary Table 2**). To construct the pYES2-VOZ2 vector, *OsVOZ2* cDNA was amplified from rice root cDNA with specific primers and inserted into the *Eco*RI/*Xho*I sites of the pYES2 vector. To co-express ACR3p-GFP *(Ura* selection; Maciaszczyk-Dziubinska et al., 2010) and OsVOZs in yeast cells, the Ura selection marker gene of pYES2-OsVOZ2 vectors was replaced with the *Kan* gene. Yeast cells transformed with rice genes and empty vectors (EVs) were selected on SG^ura−^ plates with or without G418 (0.2 mg/mL) and cultured in liquid or solid selection media supplemented with various concentrations of As(III) at 30°C for the indicated period, and their growth rates were analyzed.

### 2.3 Arsenic and cadmium tolerance test in Arabidopsis

*Arabidopsis thaliana* ecotype Columbia (Col-0) was used as the WT for all experiments. The *atvoz1* (WiscDsLox481-492O10) and *atvoz2* (SALK_115813) knockout mutants were obtained from the Sato laboratory (Nakai et al., 2013), and *atnip1;1*-1 (SALK_016617) and *atnip1;1*-2(SALK_017916) knockout seeds were purchased from Arabidopsis Biological Resource Center (ABRC) stock center. The *atvoz1 voz2* double mutant was generated by crossing *atvoz1* with *atvoz2* and selected by genomic PCR using specific primer sets (**Supplementary Table 2**). Col-0 and *atvoz1 voz2* plants were grown vertically on 1/2 Murashige and Skoog (MS) medium containing 1% (w/v) sucrose and 0.8% (w/v) Phytoagar with or without 150 µM As(V), 20 µM As(III), or 40 µM Cd(II) for 15 days under controlled conditions (16-h/8-h light/dark cycle at 22°C). Root length was measured using ImageJ software.

### 2.4 Accumulation of arsenic and metals in reproductive tissues of Arabidopsis

To analyze As and metal ions in reproductive tissues of *atvoz1* and *atvoz2* single mutants and WT, these plants were cultured in hydroponic medium for 2 weeks under short-day conditions (8-h/16-h light/dark cycle at 22°C; Gibeaut et al., 1997) and transferred to long-day conditions (16-h/8-h light/dark cycle at 22°C) to induce reproductive growth for 5 weeks. Plants exhibiting the same bolting time were selected and treated with 5 μM As(III) for 4 weeks, and As and metal ions were measured in roots, rosette leaves, stems, seed valves and seeds. To analyze As and metal ions in reproductive tissues of *atvoz1 atvoz2* double mutant and WT, first, we synchronized the flowering time between *atvoz1 atvoz2* and WT. Because the *atvoz1 atvoz2* plants exhibit a similar bolting time as the WT under short-day conditions, the plants were cultured in hydroponic medium under short-day conditions (8-h/16-h light/dark cycle) for 10 weeks and cultured in long-day conditions (16-h/8-h light/dark cycle) for 2 weeks, and stems baring flowers were cut to regenerate them at the same time. After 3 weeks, the plants were treated with 5 μM As(III) for 4 weeks, and roots, stems, seed valves, and seeds were harvested.

### 2.5 Measurement of As concentrations in Arabidopsis phloem sap

To measure As levels in phloem sap, phloem sap was collected from excised leaves as described by Tetyuk et al. (2013). Briefly, for comparison of the As concentrations in phloem sap from *atvoz1 atvoz2* and the WT, plants were cultured for 8 weeks in hydroponic medium under short-day conditions (8-h/16-h light/dark cycle) at 22°C/18°C, and subsequently treated with 5 µM As(III) for 3 days. For comparison of As concentrations in phloem sap from *atvoz1* and *atvoz2* single mutants and WT, 5-week-old plants grown on soil (12-h/12-h light/dark cycle) at 22°C were treated with 50 µM As(III) for 3 days. Rosette leaves were harvested by cutting with a razor blade at the base of the petiole, incubated in 20 mM K2-ethylenediaminetetraacetic acid (EDTA) (pH 7.0) in darkness for 1 h, and incubated in water in darkness for 8 h to extract phloem sap. The As contents in sap and rosette leaves were measured using inductively coupled plasma mass spectrometry (ICP-MS; ELAN DRC-e, PerkinElmer), and the ratio of the phloem As content and total As content of leaves was analyzed.

### 2.6 As uptake and efflux assay in Arabidopsis suspension culture cells

Arabidopsis callus formation was induced from *atvoz1 atvoz2* and WT Arabidopsis seeds in callus-inducing medium [CIM; MS salt supplemented with 3% sucrose, 2,4-D (0.5 mg/L), and kinetin (0.5 mg/L)] in agar plates. The calli were cultured in liquid CIM in a 22°C shaking incubator and subcultured every 10 days. To determine whether atvoz1 atvoz2 exhibits altered As sensitivity, the growth rates of atvoz1 atvoz2 and WT suspension cells were compared under excess As conditions. The suspension cells were treated with 5 mL of CIM supplemented with As(III) in a 22°C shaking incubator for 10 days and their fresh weights measured. For the As uptake and efflux assay, the suspension cells were treated with 100 μM As (III) in a 22°C shaking incubator for 90 min, washed with CIM, and cultured in CIM for the indicated times to analyze As release from cells.

### 2.7 Generation of transgenic rice plants

To construct the UBI1300-OsVOZ1 vector, the coding region of *OsVOZ1* was amplified via PCR with specific primers using cDNA as a template, subcloned into the pGEM-T Easy vector (Promega), and inserted into the *Eco*RI and *Xba*I sites of the UBI1300 vector (Deng et al., 2018). To analyze the tissue-specific expression of *OsVOZ1* in rice, the *OsVOZ1* promoter::GUS construct was generated. The *OsVOZ1* promoter [−2,500 to +461 base pairs (bp)] was amplified with the primer set OsVOZ1pro-F and OsVOZ1pro-R and a genomic DNA template from *Oryza sativa* (cultivar Dongjin), subcloned into the pGEM-T Easy vector, sequenced, and inserted into the *Eco*RI/*Nco*I sites of the pCAMBIA1301 vector. The constructs were introduced into *Agrobacterium tumefaciens* strain LBA4404 for plant transformation.

The Kitaake rice cultivar was used for *Agrobacterium*-mediated transformation as described previously (Jeon et al., 1999). Briefly, 4-week-old calli co-cultivated with *Agrobacterium* carrying the binary vectors were incubated on 2N6 solid medium containing hygromycin B (50 mg/L) and cefotaxime (250 mg/L) for 4 to 5 weeks for transformant selection. Hygromycin-tolerant calli were incubated on pre-regeneration medium (2N6-BA) containing hygromycin B (50 mg/L) and cefotaxime (250 mg/L) for 10 days, followed by culture on regeneration medium containing hygromycin B (25 mg/L) and cefotaxime (250 mg/L) for 4 to 6 weeks. The regenerated plants were grown in 1/2 Kimura B hydroponic medium for 2 weeks and transferred to soil. To analyze *OsVOZ1* and *OsVOZ2* expression, cDNA was synthesized from RNA isolated from UBI1300-OsVOZ1 transgenic plants, and quantitative reverse transcription PCR (qRT-PCR) was performed using a Thermal Cycler Dice Real Time System (TP-800, Takara) with gene-specific primer sets (**Supplementary Table 2**).

### 2.8 Expression analysis of genes

To identify the downstream target genes of OsVOZ1 and OsVOZ2 in yeast and Arabidopsis, expression levels of yeast genes (*ACR2*, *ACR3*, *FPS1*, *HOG1*, *HXT1*, *HXT3*, *HXT4*, *HXT5*, *HXT7*, and *YAP8*) and Arabidopsis genes (*AtNIP1;1*, *AtNIP1;2*, *AtNIP3;1*, *AtNIP5;1*, *AtNIP6;1*, *AtNIP7;1*, *AtINT2*, *AtINT4*, *AtCLT3*, *AtYSL1*, and *AtYSL3*) were analyzed by qRT-PCR using gene-specific primers as described in **Table S2**. To confirm the RNA sequence results, 14 genes showing altered expression level in *osvoz1* and *osvoz2* nodal tissues were randomly selected, and their expression levels were analyzed using RNA extracted from node samples. cDNA was synthesized from total RNA extracted from yeast cells [SM15 transformed with EV, OsVOZ1, or OsVOZ2 and treated with or without 100 µM As(III) for 3 h] and Arabidopsis plants [Col-0 and *atvoz1 atvoz2* plants treated with 100 μM As(V) for 5 h]. Total RNA was extracted from the samples using the phenol-chloroform method as described (Song et al., 2004) or TaKaRa MiniBEST Universal RNA Extraction Kit (Takara), and cDNA was synthesized using the RevertAid First-Strand cDNA Synthesis Kit (Thermo Scientific). qRT-PCR analysis was performed in the Thermal Cycler Dice Real Time System.

### 2.9 Histochemical localization of GUS

To investigate the tissue-specific expression of VOZs in Arabidopsis and rice, a histochemical assay was performed in various tissues of Arabidopsis and rice plants transformed with ProVOZ1::GUS-VOZ1, ProVOZ2::GUS-VOZ2 (Yasui et al., 2012) or OsVOZ1pro::GUS. Arabidopsis roots, stems, leaves, and siliques samples were prepared from plants during the reproductive growth stage plants grown on soil. Rice roots and leaf blades samples were prepared from 2- to 3-week-old plants grown in 1/2 Kimura B hydroponic medium, whereas node I, flower, and caryopsis samples were collected from soil-grown plants during the grain-filling stage. A *β-glucuronidase* (GUS) histochemical assay was performed as described previously (Jeon et al., 1999). All samples were fixed in 50% (v/v) ethanol:3.7% (w/v) formaldehyde:5% (v/v) acetic acid for 30 min. Node I was sectioned using a Leica VT1000 vibratome to 100-μm thickness and stained with GUS solution for 2 h to analyze GUS signals. Roots, leaf blades, flowers, and caryopsis tissues were stained with GUS solution before sectioning. After staining, the samples were dehydrated via sequential soaking in an ethanol series (50%, 70%, 90%, 95%, and 100%) and embedded in Technovit 7100 (Kulzer) as described by the manufacturer. Transverse sections (5 µm) were produced using a Leica RM2245 rotary microtome. Images were taken under a Zeiss Axiosckop2 microscope.

### 2.10 Measurement of arsenic contents

To measure As contents in yeast cells, SM15 yeast cells transforming pYES2-OsVOZ1 were cultured in SG^ura-^ supplemented with 100 µM As(III) for 6 h, harvested, and washed twice with ice-cold water. Yeast cell concentrations were measured at OD_600_. All samples (yeast and plants) were thoroughly dried at 65°C for several days. The dried samples were completely digested with 65% (w/v) HNO_3_ at 150°C, diluted in distilled water, and subjected to As content measurements using an ICP-MS.

### 2.11 RNA sequencing analysis

To prepare RNA samples, Kitaake, *osvoz1*, Dongjin, and *osvoz2* plants were cultivated in normal paddy soil, and total RNA was extracted from node I and node II of milky stage rice plants using TaKaRa MiniBEST Universal RNA Extraction Kit (Takara). RNA sequencing and data analysis were performed through Novagene sequencing service. The data were deposited into National Center for Biotechnology Information NCBI) Sequence Read Archive (SRA) (reference number: PRJNA992992). To confirm the RNA sequencing data, 14 DEGs were selected, and quantitative RT-PCR was performed using RNA of rice nodal tissues and gene specific primers sets (**Supplementary Table 2**).

### 2.12 Transactivation assay of AtVOZ2 and *AtNIP1;1* promoter

The full-length coding sequence of *AtVOZ2* and a fragment of 2,851 bp of the *AtNIP1;1* promoter were cloned into the donor vector pENTR™/D-TOPO™ (Invitrogen, Gaithersburg, MD, USA). *AtVOZ2* was then introduced into p2GW7 carrying the full CaMV 35S promoter (Karimi et al., 2002) by LR cloning (Invitrogen, Gaithersburg, MD, USA). The AtNIP1;1::LUC (firefly luciferase) construct was developed by introducing the *AtNIP1;1* promoter upstream of *LUC* of a modified LUC-pUC19 vector. The UBI pro::GUS vector was used as an internal control. The effector (35Spro::AtVOZ2), reporter (AtNIP1;1::LUC), and internal control (UBI pro::GUS) vectors were co-transfected into rice protoplasts derived from suspension-cultured Oc cells established from seedling roots of indica cultivar C5928 (Baba et al., 1986). Luciferase activity was measured using Luciferase Assay Systems (Promega, USA) following the manufacturer’s instruction. GUS and luciferase (LUC) activities were recorded using a Wallac Victor2 1420 Multilabel Counter (Conquer Scientific, USA).

### 2.13 Statistical analyses

To analyze the statistical significance of the data, a one-way analysis of variance (ANOVA; significance level of P < 0.05) was performed using GraphPad Prism 4, or two-tailed Student’s *t*-tests (significance level of *P < 0.05 or **P < 0.01) were performed using Microsoft Excel.

## 3 Results

### 3.1 Identification of novel arsenic tolerance genes using a rice cDNA library

We developed a yeast expression system to identify novel rice genes conferring As tolerance related with the PC pathway (**Supplementary Figure 1**). Therefore, we used the SM15 yeast strain, an As-sensitive yeast strain expressing the wheat PC synthase gene (TaPCS1). Briefly, we constructed a cDNA library using mRNA extracted from roots of rice plants grown in medium containing As. We then introduced the library into the yeast strain SM15, and selected yeast colonies that grew in medium containing As. We identified 25 genes that increased As tolerance in yeast using this approach (**Supplementary Figure 1**, **Supplementary Table 1**). They encoded for proteins playing diverse roles, including transcriptional regulation, protein synthesis and modification, secondary metabolic processes, signal transduction, transport activity, autophagy, and hydrolases. *VOZ1* (*Vascular plant One-Zinc finger1* transcription factor), polyubiquitin, and epimerase genes were the most frequently found genes increasing As tolerance of yeast clones (VOZ1: 32/384, polyubiquitin: 90/384, epimerase: 81/384). Interestingly, genes involved in protein degradation processes, such as polyubiquitin, 26S proteasome complex subunit, AAA-ATPase, and autophagy-related protein 8A, were also identified in this screen (**Supplementary Table 1**). The result suggests that the proteolysis pathway mediated by ubiquitination, autophagy, and 26S proteasome is an important mechanism to rescue yeast cells from As toxicity. It has been described that As induces inactivation and misfolding of proteins harboring amino acid residues with sulfur by binding them, and expression of genes involved in ubiquitination and autophagy enhances As tolerance through the degradation of unfolded proteins or As importers in yeast cells (Guerra-Moreno et al., 2015; Jochem et al., 2019).

### 3.2 OsVOZ1 and OsVOZ2 transcription factors enhance As tolerance by enhancing ACR3 protein amount in budding yeast

**Figure 1:**
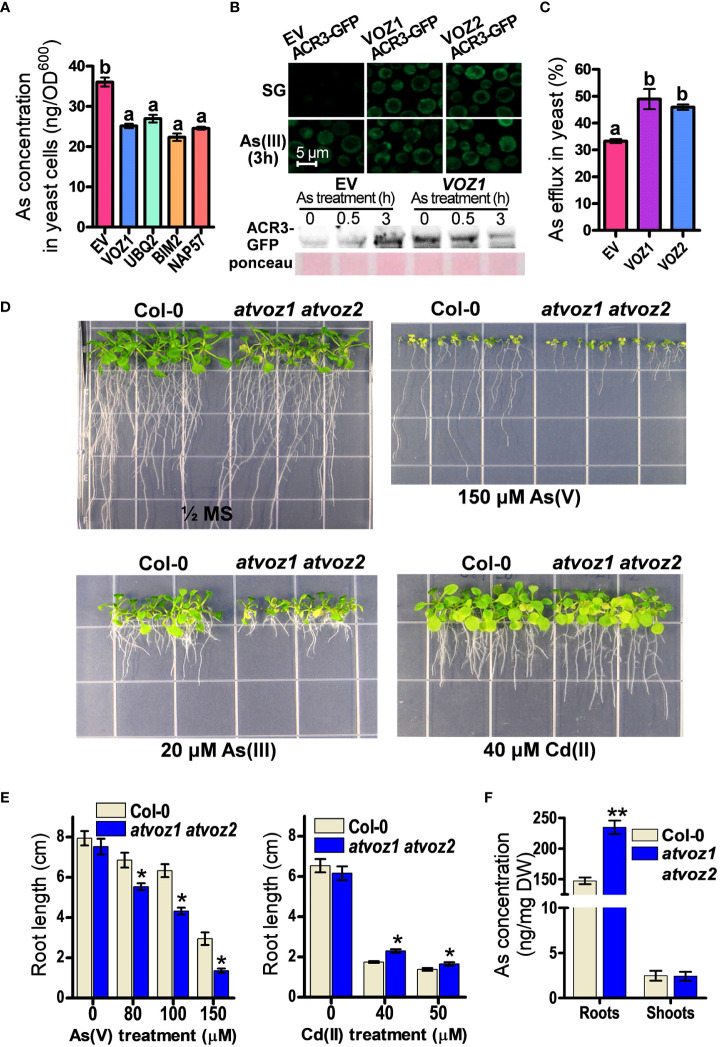
Plant VOZs contribute to As tolerance in yeast and Arabidopsis. **(A)** As concentrations in yeast cells heterologously expressing As tolerance rice genes. As contents were measured in yeast cells treated with 100 μM As(III) for 6 h (n = 5). **(B)** Analysis of GFP fluorescence and amount of ACR3-GFP in yeast cells co-transformed with *ACR3p-GFP* and empty vector (EV), *OsVOZ1*(*VOZ1*), and *OsVOZ2*(*VOZ2*). **(C)** As efflux in yeast cells transformed with EV, *OsVOZ1*, and *OsVOZ2*. Yeast cells were treated with As(III) for 30 min and applied to normal medium to release As (n = 5). **(D)** Phenotypic comparison of WT and *atvoz1 atvoz2* mutant in media containing As or Cd. **(E)** Root growth in media containing As or Cd of Col-0 and *atvoz1 atvoz2* plants. Plants were grown on ½ MS medium agar plates supplemented with 150 µM As(V), 20 µM As(III), or 40 µM Cd(II) for 15 days (n = 30). **(F)** As concentration in WT and *atvoz1 atvoz2*. Plants were grown in hydroponic medium for 5 weeks and then exposed to 5 μM As(III) for 7 days (n = 5). Lines and bars indicate means ± SE. The statistical significance was calculated using one-way ANOVA test (*P* < 0.05) and Student’s *t-*test (*P < 0.05 and **P < 0.001). Different letters above bars represent significant difference.

Among the candidate genes, *OsVOZ1* conferred the highest As tolerance to SM15 yeast cells, but it did not enhance Cd tolerance in *ycf1* yeast cell (SM14) (**Supplementary Figures 2A, B**). To investigate whether OsVOZ1 regulates As sequestration or As efflux to improve As tolerance in yeast, we compared As concentrations in yeast cells transformed with *OsVOZ1* and other candidate genes versus the EV. To avoid differences in As concentrations due to growth differences, we limited the As treatment to 6 h and a concentration of 100 μM As(III), as this treatment did not cause any noticeable growth difference between the yeast lines during the defined time. In yeast cells expressing *OsVOZ1*, the As(III) concentration was reduced by 29% compared with the EV control (**Figure 1A**). The other yeast cells expressing candidate genes found in our screen, including genes for polyubiquitin (UBQ2), the BIM2 transcription factor, and NAP57, accumulated also less As than the EV control (**Figure 1A**). These results suggest that the putative As tolerance genes identified in this study, including *OsVOZ1*, confer As tolerance in yeast by inhibiting As(III) accumulation due to decreased uptake or increased efflux.

PCs highly affect the cytosolic As(III) mobility in Arabidopsis (Liu et al., 2010). However, complexes of As and PCs are unstable in neutral pH(cytosol) but stable in low pH (vacuolar pH). Therefore, cytosolic As can still attack important molecules in cell even in the presence of PCs, if As-PCs are not compartmented into vacuole by PCs-As transporters (Song et al., 2010; Song et al., 2014). To know whether the Cd tolerance shown in SM15 yeast expressing *OsVOZ1* is related to PCs, we compared the growth rates of SM14 (quadruple mutants of C-type of ABC transporter including YCF1) and SM15 [SM14 expressing *TaPCS1* (*Triticum aestivum Phytochelatin Synthase1*) driven by the cooper inducible promoter *pCUP1*] yeast cells (Mendoza-Cózatl et al., 2010) transformed with *OsVOZ1* or *OsVOZ2*, *a OsVOZ1* homolog. However, the yeast cells exhibited comparable growth rates under As treatment (**Supplementary Figure 2B**), indicating that the presence of PCs is not required for the roles of OsVOZ1 and OsVOZ2 in As tolerance in yeast.

To elucidate molecular mechanisms how OsVOZ1 and OsVOZ2 affect As tolerance by reducing of As concentration in yeast cell (**Figure 1A**), we first analyzed the transcript levels of a large number of genes known to participate in As tolerance, uptake, and efflux in the yeast system to identify genes whose expression levels are regulated by OsVOZ1 and OsVOZ2: As(III) major uptake transporters (HXTs and FPS1; Liu et al., 2004; Maciaszczyk-Dziubinska et al., 2010), As(III) efflux transporter located at plasma membrane (ACR3; Bobrowicz et al., 1997; Wysocki et al., 1997; Ghosh et al., 1999), As(V) reductase (ACR2; Bobrowicz et al., 1997), transcription factor of As metabolism (YAP8; Di and Tamás, 2007), and mitogen-activated protein kinase (MAPK) regulating As transporters (HOG1; Jochem et al., 2019). Except *HXT9*, the transcript levels of *ACR3*, *FPS1*, *HOG1*, and *HXTs* were not significantly altered by *OsVOZ1* and *OsVOZ2* expression (**Supplementary Figure 2C**). However, the knockout of *HXT9* did not enhance As(III) tolerance (**Supplementary Figure 2D**), suggesting the reduced expression of *HXT9* is not the cause of As tolerance mediated by *VOZs*.

As(III) extrusion from the cell into the medium mediated by the As(III) efflux transporter ACR3 is critical for As tolerance in budding yeast (Wysocki et al., 1997; Ghosh et al., 1999; Zhou et al., 2009). Therefore, we analyzed As tolerance in the *acr3 ycf1* yeast strain expressing OsVOZ1 and OsVOZ2, to examine whether the reduced As accumulation in yeast expressing OsVOZ1 or OsVOZ2 requires the presence of the As efflux transporter gene ACR3, although OsVOZ1 and OsVOZ2 did not alter transcription level of *ACR3*. Unexpectedly, OsVOZ1 and OsVOZ2 did not enhance As tolerance in these yeast cells (**Supplementary Figure 2B**), suggesting that As tolerance mediated by OsVOZ1 and OsVOZ2 depends on the As(III) efflux transporter ACR3. To verify whether OsVOZs enhance the protein level of ACR3 by posttranscriptional regulation to reduce cytosolic As concentration, we compared fluorescence intensity of ACR3-GFP in yeast cells co-transformed with ACR3p-GFP and OsVOZ1, OsVOZ2, or EV. In the absence of As(III), *ycf1 acr3* yeast mutant cells transformed with *OsVOZ1* or *OsVOZ2* exhibited bright GFP fluorescence of ACR3p-GFP in the cytosol and at the plasma membrane and ACR3-GFP protein band, whereas cell transformed with the EV showed a dot pattern of ACR3p-GFP with dim GFP fluorescence and faint protein band of ACR3-GFP. In the presence of As(III), all yeast lines showed bright GFP fluorescence and protein bands of ACR3-GFP (**Figure 1B**). Furthermore, expression of *OsVOZ1* and *OsVOZ2* enhanced As efflux activity during a short-term period (loading of As for 30 min and then effluxing it for 30 min) (**Figure 1C**). These results indicate that OsVOZ1 and OsVOZ2 might confer As tolerance to yeast through the activation of ACR3 via post-transcriptional modification.

### 3.3 Loss of function of *AtVOZ1* and *AtVOZ2* causes increased sensitivity to As in Arabidopsis

AtVOZ1 and AtVOZ2 exhibit a high–amino acid sequence similarity with OsVOZ1 and OsVOZ2 (Mitsuda et al., 2004; **Supplementary Figure 3**). They have conserved regions on the N-terminal and C-terminal domains. The C-terminal domain contains a zinc finger motif (CCCH). To examine whether the loss of function of Arabidopsis *VOZ1* and *VOZ2* alters As tolerance in Arabidopsis, we identified the corresponding knockout plants and created the corresponding double mutant. First, we compared growth rates of *atvoz1* and *atvoz2* single mutants and WT plants under normal or As excess conditions. The single mutants exhibited similar growth patterns with WT in both conditions supplemented with or without As(V) (**Supplementary Figure 4**). In other work on VOZs, it was shown that the mutants behaved similarly as those chosen in the present work (Nakai et al., 2013; Kumar et al., 2018), because AtVOZ1 and AtVOZ2 have been shown to be functionally redundant (Yasui et al., 2012). In contrast to single mutants, the *atvoz1atvoz2* double-mutant plants produced smaller leaves and an early senescence phenotype in their cotyledons compared with the WT under normal growth conditions (**Figure 1D**). When exposed to As(V) or As(III), the *atvoz1 atvoz2* double-knockout mutant had much shorter primary roots than the WT (**Figures 1D, E**). These results suggest that both AtVOZ1 and AtVOZ2 play important roles in As tolerance of Arabidopsis and exhibit redundant functions related to As tolerance. We also examined whether the *atvoz1 atvoz2* mutant is sensitive to other heavy metals. The *atvoz1 atvoz2* exhibited slightly longer primary roots than the WT when Cd(II) was present in the medium (**Figures 1D, E**), but *atvoz1* and *atvoz2* single mutant did not exhibit altered Cd tolerance (**Supplementary Figure 4**). However, the *atvoz1 atvoz2* mutant was hypersensitive to Fe(II) and Zn(II), whereas the mutant showed a comparable growth to the WT in the presence of excess Cu(II) (**Supplementary Figure 5**). These results suggest that AtVOZ1 and AtVOZ2 differentially affect the responses to As and heavy metal treatment in Arabidopsis and in yeast. To understand the reason why the *atvoz1 atvoz2* mutant is sensitive to As, we analyzed As concentrations in 5-week-old vegetative plants treated with As for 7 days. The roots of *atvoz1 atvoz2* plants accumulated significantly more As than those of the WT, but shoots of the mutant accumulated similar amounts of As to those of WT (**Figure 1F**). However, there was no difference of As extrusion activity from the roots of *atvoz1 atvoz2* and WT (**Supplementary Figure 6**). These results suggest that the As hypersensitivity in the double mutant might be originated from the accumulation of As by increased As uptake transport activity in the mutant roots.

### 3.4 AtVOZ1- and AtVOZ2-dependent As accumulation in different Arabidopsis tissues

**Figure 2:**
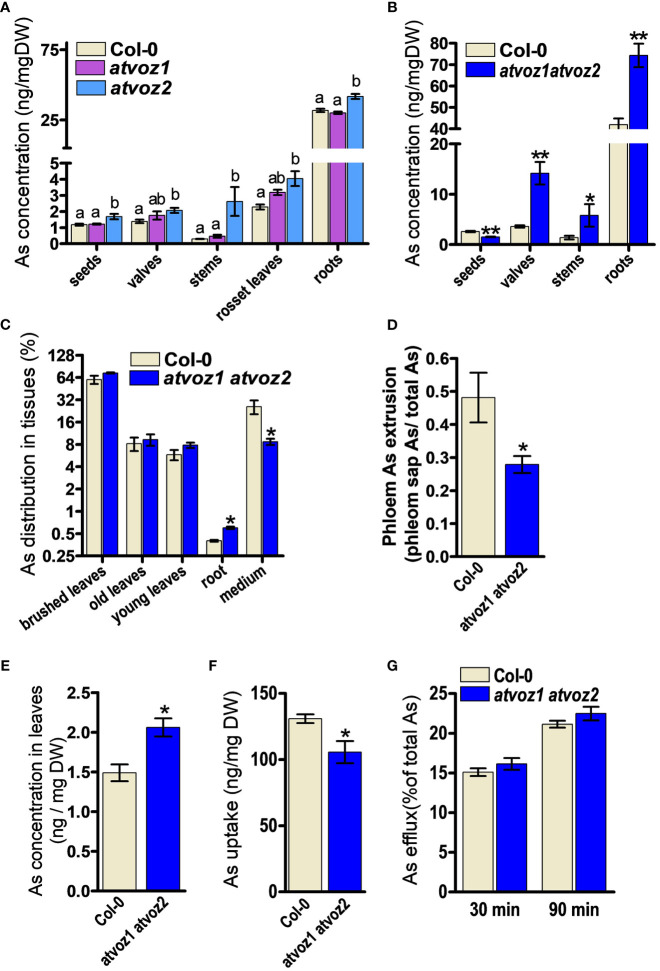
Concentrations of As in various tissues and phloem sap from atvoz1, atvoz2 single mutants, *atvoz1 atvoz2* double mutant, and wild-type plants. **(A)** As concentration in various tissues of atvoz1 and atvoz2 mutants. Seven-week-old Arabidopsis plants grown in hydroponic medium were treated with 5 μM As(III) for 4 weeks (n = 6 plants). **(B)** As concentration in various tissues of *atvoz1 atvoz2* double mutants. Fifteen-week-old Arabidopsis plants grown in hydroponic medium were treated with 5 μM As(III) for 4 weeks (n = 6 plants). **(C)** As leaf feeding assay. WT and *atvoz1 atvoz2* mutant were grown in hydroponic medium for 8 weeks, supplemented with 50 µM As(V) through leaves for 5 days, and then keep them in the medium for 5 days to induce As translocation (n = 5). **(D)** Phloem As extrusion activity in *atvoz1 atvoz2* double mutant and WT Arabidopsis plants. Eight-week-old Arabidopsis plants cultured in hydroponic medium were treated with 5 µM As(III) for 3 days, and phloem exudates were collected using the K2-EDTA method (n = 10 plants). **(E)** Concentrations of As in leaves from 8-week-old hydroponic cultured Arabidopsis plants treated with 5 µM As(III) for 3 days (n = 10 plants). **(F)** As(III) uptake in suspension cultured cells of WT and *atvoz1 atvoz2*. Cells were incubated in callus culture medium containing 100 μM As(III) for 90 min, washed with callus culture medium, and measured As concentration in the cells (n = 6 culture). **(G)** As(III) release from suspension cultured cells of WT and *atvoz1 atvoz2*. Cells were incubated in callus culture medium containing 100 μM As(III) for 90 min, washed with callus culture medium, and incubated in callus culture medium without As(III) for 30 and 90 min to measure As efflux (n = 6 culture). Bars indicate means ± SE. The statistical significance was calculated using one-way ANOVA test (*P* < 0.05) and Student’s *t-*test (*P < 0.05 and **P < 0.001). Different letters above bars represent significant difference.

Arabidopsis *VOZ1* and *VOZ2* are highly expressed in the vasculature throughout the entire developmental stage of the plant (Yasui et al., 2012). We therefore analyzed As concentrations in various tissues of *atvoz1* and *atvoz2* single and *atvoz1 atvoz2* double mutants to determine whether the loss of function of *AtVOZ1* and *AtVOZ2* alters As accumulation. We treated hydroponic cultured 7-week-old single mutants and WT with 5 μM As(III) for 4 weeks; harvested seeds, seed valves, stem, leaves, and roots; and measured As. Plants at the same bolting stage were chosen for this experiment. The *atvoz1* and *atvoz2* single mutants did not exhibit altered As concentration in seeds. In contrast, slightly enhanced As concentration could be observed in leaves, whereas, in stems, a more than nine-fold increase of As could be observed in the *atvoz2* compared with the WT (**Figure 2A**). These results suggest that AtVOZ2 was mainly responsible for metal(loid) translocation from roots to shoots, whereas the role of AtVOZ1 might be quite moderate. We also compared metals and As concentrations in the *atvoz1 atvoz2* double mutant and WT (**Figure 2B**). We grew plants in hydroponic medium under short-day conditions to synchronize flowering time between *atvoz1 atvoz2* and WT and treated plants having the same bolting stage with As(III), because the double mutant exhibits delayed flowering time compared with the WT under long-day conditions (Yasui et al., 2012). *atvoz1 atvoz2* seeds accumulated only 58% of As compared with the WT, but this double mutant accumulated much more As in seed valves, stems, and roots of 395%, 418%, and 177% of the WT, respectively (**Figure 2B**). We compared the As concentrations of various tissues of Col-0 (WT) cultured under short-day and long-day conditions, to know whether As accumulation is changed by the different conditions. However, the As concentrations of WT plants cultured long-day and short-day conditions were similar (**Figures 2A, B**). To further understand the roles of AtVOZ1 and AtVOZ2 for As transport, we examined As translocation via the phloem pathway using an As leaf feeding assay. Notably, 25% of As taken by leaves was secreted into the medium of cultured WT plants, whereas only 8% of total As was detected in the medium cultured *atvoz1 atvoz2* plants. The mutant roots accumulated more As than those of WT (**Figure 2C**). These results suggest that AtVOZ1 and AtVOZ2 affect the As translocation from shoots to roots via the phloem pathway and that As accumulation in roots and shoots of *atvoz1 atvoz2* might be caused by reduced As secretion into the culture medium.

### 3.5 The at*voz1* at*voz2* double mutant has a reduced As concentration in the phloem sap

To determine whether altered As distribution in stem, leaves, and seeds of *atvoz2* and *atvoz1 atvoz2* plants is due to altered metal(loid) transport in the phloem, which represents the major pathway of As redistribution into various organs including seeds, we analyzed the metal(loid) contents in phloem exudates collected from the rosette leaves using the K-EDTA methods (Tetyuk et al., 2013). During the experiment, leaves were kept in the dark and at high humidity to minimize transpiration. In *atvoz1 atvoz2* plants, As concentrations were significantly lower in the phloem sap than in the WT (**Figure 2D**), whereas total As concentration of leaves from *atvoz1 atvoz2* plants treated with 5 µM As(III) was slightly higher than that of the WT (**Figure 2E**). In the phloem sap, the concentration of Mn and Zn of *atvoz1 atvoz2* plants were much lower than those of WT, but Cu concentration was not different (**Supplementary Figures 7A–C**). In leaves, total concentrations of Cu, Mn, and Zn were not different between the two lines (**Supplementary Figures 7D–F**). To verify whether genes involved in phloem transport of As, Mn, and Zn are altered in atvoz1 atvoz2 mutant, we analyzed the expression of *AtINT2* and *AtINT4* (As phloem transporter) and of AtYSL1 and AtYSL3 (Zn phloem transporter). The expression levels of *AtINT2* and *AtINT4* were not highly changed in *atvoz1atvoz2* mutant (**Supplementary Figures 7G, H**), but the transcripts of *AtYSL1* and *AtYSL3* were reduced in the mutant (**Supplementary Figures 7I, J**). These results indicate that transport of As, Mn, and Zn through the phloem is reduced in *atvoz1 atvoz2* plants, perhaps because AtVOZ1 and AtVOZ2 regulate the expression of transporters responsible for the phloem loading or unloading of As, Mn, and Zn.

To investigate the reason for the reduced As concentration in the phloem sap of *atvoz1 atvoz2* at the cellular level, we generated calli from *atvoz1 atvoz2* and WT Arabidopsis seeds, grew them in suspension culture, and subjected them to As uptake and efflux assays. The *atvoz1 atvoz2* cells took up less As than the WT but did not show enhanced As efflux activity, indicating reduced loading of As (**Figures 2F, G**). Together, these results suggest that *atvoz1 atvoz2* cells contain either less As transporters or As transporters with reduced activity compared with WT cells. In addition, the reduced As phloem loading in *atvoz1 atvoz2* might lead to reduce As concentrations in its seeds.

### 3.6 VOZ1 and VOZ2 are expressed in phloem and xylem parenchyma cells in rice and Arabidopsis

**Figure 3:**
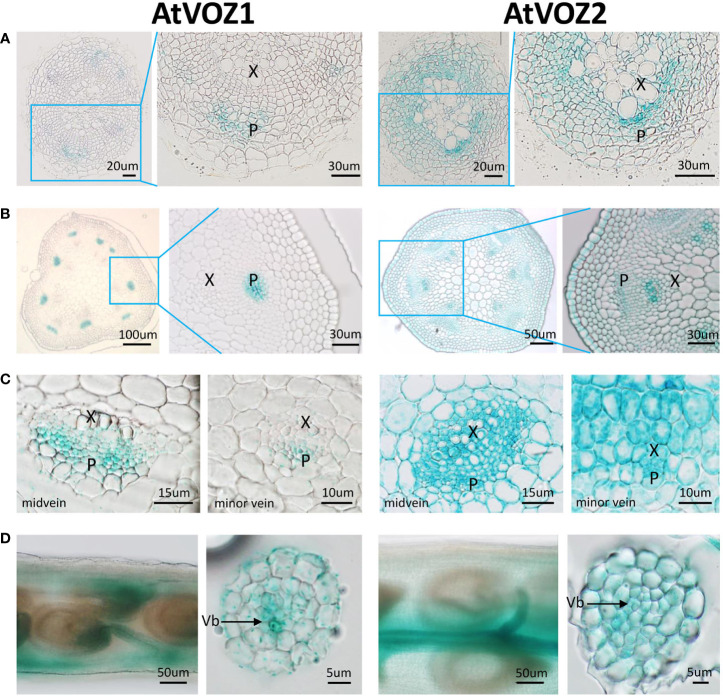
Comparison of tissue-specific expression patterns of *AtVOZ1* and *AtVOZ2* in *ProVOZ1::GUS-VOZ1* and *ProVOZ2::GUS-VOZ2* Arabidopsis plants. Roots **(A)**, stems **(B)**, leaves **(C)**, and siliques **(D)** were harvested at the reproductive stage of Arabidopsis plants expressing *ProVOZ1::GUS-VOZ1* and *ProVOZ2::GUS-VOZ2*, stained with GUS staining solution, and cross-sectioned to 5-μm thickness. X, xylem; P, phloem; Vb, valve bundle.

**Figure 4:**
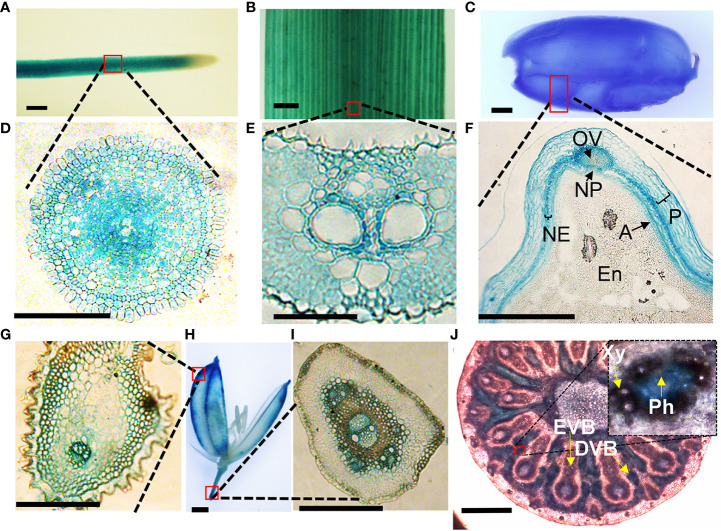
Tissue-specific expression of *OsVOZ1p::GUS*. Roots **(A, D)**, leaf blades **(B, E)**, caryopsis **(C, F)**, and mature rice florets **(G, H, I)** were stained with GUS staining solution and cross-sectioned to 5-μm thickness, and nodes I **(J)** were cross-sectioned to 100-μm thickness and stained with GUS solution for 2 h. A, aleurone layer; En, endosperm; NE, nucellar epidermis; NP, nucellar projection; OV, ovular vascular trace; P, pericarp; EVB, enlarged vascular bundle; DVB, diffuse vascular bundle; Xy, xylem; Ph, phloem. Scale bars = 500 μm **(A–D, H, J)** and 100 μm **(E–G, I)**.

To get clues on how VOZs regulate As tolerance and accumulation in Arabidopsis and rice, we monitored the tissue-specific expression of *VOZ1 or VOZ2* in rice and Arabidopsis plants transformed with *ProVOZ1::GUS-VOZ1*, *ProVOZ2::GUS-VOZ2*, and *OsVOZ1pro::GUS* constructs. In *ProVOZ1::GUS-VOZ1* and *ProVOZ2::GUS-VOZ2* transgenic Arabidopsis plants, GUS signals were primarily detected in the vasculature of roots, stems, leaves, septa, and funiculi (**Figure 3**). *GUS-VOZ1* was specifically expressed in phloem cells of roots, leaves, and stems (**Figure 3**, left panel), whereas the GUS-VOZ2 signals were detected in phloem as well as xylem parenchyma cells of these tissues and stems and, the most pronounced GUS signals were detected in xylem parenchyma cells (**Figure 3**, right panel), suggesting that *AtVOZ1* and *AtVOZ2* might be expressed specifically in different tissues. *AtVOZ1* and *AtVOZ2* were also highly expressed at the funiculus vasculature of siliques, an important tissue for solute allocation to the seeds (**Figure 3D**). In rice plants expressing *OsVOZ1pro::GUS*, GUS signals were also present in the vasculature of various tissues participating in As transport, such as roots, leaves, caryopsis, flowers, and node I (**Figures 4A–J**). Strong GUS signals were detected in the oval vasculature of the caryopsis (**Figure 4F**), with additional GUS signals in phloem cells of the EVB, DVB, and nodal vascular anastomosis (NVA) of node I (**Figure 4J**). The vasculature tissue specific expression of Arabidopsis and rice *VOZ1* and *VOZ2* suggests that both genes might have a common role in regulating genes involved in solute transport that translocate As into grains, but their targets might be different.

To investigate whether Arabidopsis and rice VOZs are induced to express in response to external As, we analyzed their expression patterns in Arabidopsis and rice plants treated with As using qRT-PCR. However, their transcript levels did not change in response to As (**Supplementary Figure 8**).

### 3.7 Altered expression levels of *NIP1;1* in *atvoz1 atvoz2* plant enhanced As sensitivity and As accumulation

**Figure 5:**
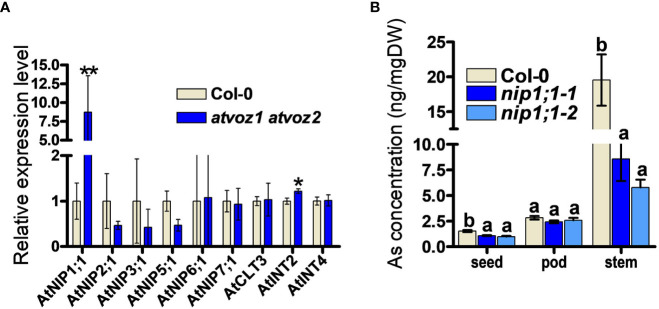
As concentrations in tissues from knockout plants of *AtNIP1;1*, exhibiting enhanced expression levels in *atvoz1 atvoz2* mutant. **(A)** Quantitative RT-PCR of genes involved in As transport in Arabidopsis. The expression level of each gene was normalized to *AtTUB8*. Bars indicate means ± SD (Biological replicates n = 3). **(B)** As concentrations in tissues from *atnip1;1* knockout and Col-0 plants. Samples were prepared from 11-week-old Arabidopsis plants treated with 5 μM As(III) for 4 weeks. Bars indicate means ± SE (n = 8 plants). The statistical significance was calculated using Student’s *t-*test (*P < 0.05 an **P < 0.001) and one-way ANOVA test (*P* < 0.05). Different letters above bars represent significant difference.

To identify possible downstream targets for As tolerance and As distribution mediated by VOZ1 and VOZ2 in Arabidopsis, we searched for candidates downstream of AtVOZ1 and AtVOZ1 in a RNA micro array and RNA-seq datasets performed using *atvoz1 atvoz2* plants (Nakai et al., 2013; Kumar et al., 2018; Prasad et al., 2018). We could find a putative As transporter genes among the differentially expressed (DE) genes in the datasets in this double mutant: The expression level of *AtNIP7;1* was increased compared with its WT (Prasad et al., 2018). AtNIP7;1 works as As(III) uptake transporter in Arabidopsis and yeast, and the loss of function in AtNIP7;1 led to increased plant tolerance to As(III) and reduced As concentration in seeds (Isayenkov and Maathuis, 2008; Lindsay and Maathuis, 2016). However, we could not confirm the altered expression of *AtNIP7;1* under our experimental conditions (**Figure 5A**). Because the *atvoz1 atvoz2* mutant exhibited As hypersensitivity and altered As accumulation in various tissues, we compared the expression levels of known genes related to As transport, such as genes encoding aquaporins (AtNIP1;1, AtNIP1;2, AtNIP3;1, AtNIP6;1; Kamiya et al., 2009; Xu et al., 2015) and inositol transporters participating in As seed accumulation (*AtINT2* and *4*; Duan et al., 2016). However, we did not include *ABCC*s, because the phenotype of *ABCC*s knockout mutants of Arabidopsis and rice are different from those of the *vozs*. The *atabcc1 atabcc2* is sensitive to As and Cd (Song et al., 2010; Park et al., 2012), whereas *atvoz1 atvoz2* was not sensitive to Cd and exhibited lower seed As compared with its WT. Notably, the expression level of *AtNIP1;1* was higher in *atvoz1 voz2* than in the WT by a factor of 7 (**Figure 5A**). The loss of function of *AtNIP1;1* causes reduced As(III) tolerance and increased As accumulation in roots of *Arabidopsis thaliana* (Kamiya et al., 2009) as similar to *atvoz1 atvoz2* (**Figure 1**). To determine whether *AtNIP1;1* contributes to the distribution of As in various tissues of Arabidopsis as the downstream target of *atvoz1 atvoz2*, we compared As concentration in seeds, pods, and stems from *atnip1;1* and Col-0. As concentration of seeds and stems were highly decreased in two alleles of *atnip1;1* mutant compared with Col-0 (**Figure 5B**). The reduced As accumulation in seeds of *atnip1;1* was similar to that of *atvoz1 atvoz2* exhibiting a reduced As accumulation compared with their WT, but As concentration in stems of *atnip1;1* mutants was opposite to that of *atvoz1 atvoz2* (**Figures 2B**, **5B**). However, the luciferase activity of AtNIP1;1pro::LUC was not activated by AtVOZ2 in rice cells transformed with 35Spro:: AtVOZ2 and AtNIP1;1pro:: Luc vectors (**Supplementary Figure 9**). These results suggest that *AtNIP1;1* is not directly regulated by AtVOZ2 and that the enhanced expression levels of *AtNIP1;1* in *atvoz1 atvoz2* might cause the altered As tolerance and accumulation in seeds.

### 3.8 Reduced *OsVOZ1* and *OsVOZ2* expression decreased As accumulation in rice grains

**Figure 6:**
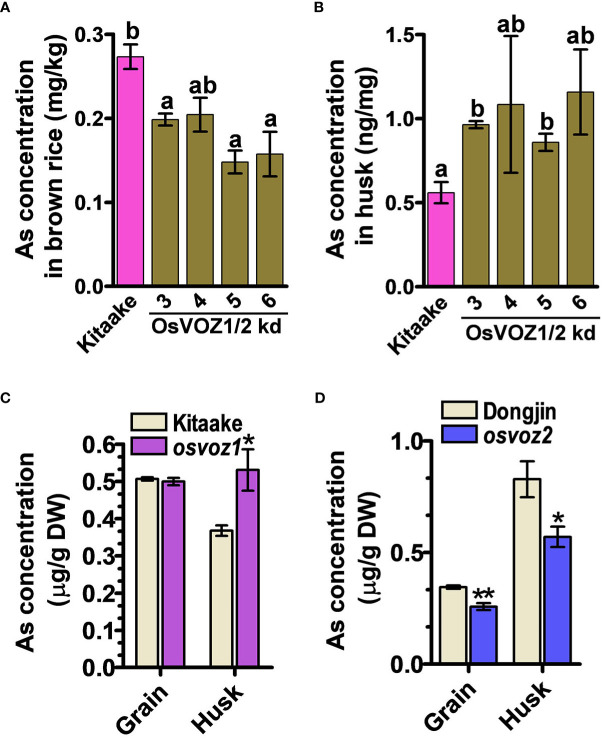
As concentrations in *OsVOZ1 OsVOZ2* knockdown and *OsVOZ1* and *OsVOZ2* single knockout mutants. Rice plants were cultivated in normal paddy soil, and As concentrations in brown rice and husk from *OsVOZ1 OsVOZ2* double knockdown were determined (OsVOZ1/2 kd; **(A, B)**, *osvoz1* and *osvoz2* single knockouts **(C, D)** were compared with those of their corresponding WTs. Bars indicate means ± SE (n = 5 plants). The statistical significance was calculated using one-way ANOVA test (*P < 0.05) and Student’s *t*-test (*P < 0.05 and **P < 0.001). Different letters above bars represent significant difference.

Because the *osvoz1 osvoz2* double mutant has been shown to be lethal (Wang et al., 2021), we decided to explore the functions of OsVOZs in rice by producing an overexpressing plant. Therefore, we transformed rice plants with *UB1300::OsVOZ1*. Unexpectedly, the transgenic lines exhibited a reduced expression of endogenous *OsVOZ1* and *OsVOZ2* (**Supplementary Figure 10A**), possibly due to a co-suppression event (Napoli et al., 1990). Endogenous *OsVOZ1* and *OsVOZ2* transcripts were present at levels of only 0.5% to 1% and 20% to 40%, respectively, in transgenic versus WT plants. Similar to the Arabidopsis *voz1voz2*, the growth of *UBI1300::VOZ1* T3 transgenic rice plants was also impaired (**Supplementary Figure 10B**, right). However, the rice transgenic plants did not exhibit hypersensitivity in the presence of excess of As(III) (**Supplementary Figure 10B**, left). When cultivated in normal paddy soil, seeds of *UBI1300::*VOZ1 plants contained much less As than WT; the brown rice grains of the transgenic plants accumulated only 47%–74% of that determined in the WT (cultivar Kitaake) (**Figure 6A**), whereas the transgenic plants accumulated more As in husks than did WT plants (**Figure 6B**). The single knockout of *OsVOZ1* and *OsVOZ2* also exhibited altered As accumulation in brown rice and husk; the *osvoz1* accumulated more As in husks, whereas *osvoz2* exhibited reduced As accumulation in brown rice and husk (**Figures 6C, D**). To identify As transporters affected by OsVOZs in rice node I, a critical tissue regulating the As accumulation in grains, we performed RNA sequencing analysis in node I samples of *osvoz1*, *osvoz2*, and their isogenic WTs. A total of 1,029 genes were DE in *osvoz2* mutant, and expression of 184 genes was altered in *osvoz1* mutant (**Supplementary Table 3**). However, transcription levels of genes participating As transport such as aquaporins, phosphate transporters, and ABCC1 were not found in DEG lists. The result implies that OsVOZs might regulate the grain As accumulation through the post-translational modification of As transporters. The result of RNA sequencing was confirmed by quantitative RT-PCR using 14 different genes showing altered expression in nodal tissues of *osvoz1* and *osvoz2* (**Supplementary Figure 11**).

## 4 Discussion

### 4.1 Many rice genes improve As tolerance in yeast

To identify the mechanisms and genes involved in As tolerance in rice roots, we screened for rice genes that improve As tolerance in yeast cells through the expression of a cDNA library generated from rice roots exposed to As. Genes involved in the regulation of transcription and post-transcriptional modification, especially polyubiquitin, 26S proteasome complex subunit, and autophagy were found at high frequency (**Supplementary Figure 1**, **Supplementary Table 1**). Ubiquitination has been shown to enhance As tolerance in living cells through the degradation of misfolded proteins triggered by As(III) covalent binding into the thiol groups of the corresponding proteins (Yen et al., 2005; Zhang et al., 2010; Shen et al., 2013). Furthermore, ubiquitination can also regulate directly, or indirectly key proteins related to As transport in yeast (As uptake, efflux, and vacuolar sequestration) such as FPS1, YCF1, ACR3, and HXTs, to protect cells from As toxicity (Yen et al., 2005; Di and Tamás, 2007; Gulshan et al., 2012; Jochem et al., 2019). In plants, ubiquitin-proteasome pathway is also involved in As tolerance (Zhang et al., 2023). Arabidopsis ARS5, a component of the 26S proteasome complex, negatively regulates thiol biosynthesis and As tolerance in Arabidopsis (Sung et al., 2009). Rice OsHIR1, a RING-type ubiquitin E3 ligase, degrades the tonoplast intrinsic protein 4;1 OsTIP4;1 via the ubiquitin 26S proteasome, and overexpression of OsHIR1 enhances As tolerance in Arabidopsis (Lim et al., 2014). OsVOZ1 and OsVOZ2 are degraded through the ubiquitin-26S proteasome pathway (Wang et al., 2021). Here, we found that the increased As tolerance mediated by OsVOZ1 and OsVOZ2 requires ACR3 transporter in yeast (**Supplementary Figure 2B**), but heterologous expression of OsVOZ1 and OsVOZ2 did not change the expression levels of *ACR3* (**Supplementary Figure 2C**). However, OsVOZ1 and OsVOZ2 enhanced fluorescence signal from ACR3-GFP and As(III) efflux in yeast in short term period (**Figures 1B, C**). These results suggest that both transcription factors might indirectly induce As tolerance in yeast cells by regulating pathways for the efflux of As(III) via post-transcriptional modification (**Supplementary Figure 12A**).

### 4.2 VOZs are required for As tolerance and As translocation in various tissues

VOZ proteins are plant-specific zinc finger–type transcriptional activators that are highly conserved throughout land plants. VOZ1 and VOZ2 were first identified as binding proteins that specifically recognize the GCGTNx7ACGC sequence of a 38-bp cis-acting region of *AVP1* in Arabidopsis. AVP1 is a vacuolar H^+^-pyrophosphatase that functions during pollen development (Mitsuda et al., 2004). Arabidopsis *VOZ* genes confer abiotic and biotic stress tolerance and play important roles in flower development (Yasui et al., 2012; Nakai et al., 2013; Prasad et al., 2018). The *atvoz1 atvoz2* double mutant exhibits delayed seed germination, retarded growth, reduced salt tolerance, and decreased expression levels of salt-inducible genes compared with the WT (Prasad et al., 2018), suggesting that VOZ transcription factors act as positive regulators of several salt-responsive genes. However, so far it is not known which downstream target genes of AtVOZ1 and AtVOZ2 are responsible for the tolerance to the abiotic stresses, including drought and excess salt (Nakai et al., 2013; Prasad et al., 2018). Here, we found that *atvoz1 atvoz2* plants were more sensitive to As and accumulated more As in roots but less As in seeds compared with the WT (**Figures 1D, E**, **2B**). Furthermore, the expression level of the As uptake transporter gene, *AtNIP1;1* was negatively affected by AtVOZ1 and AtVOZ2 (**Figure 5A**). In addition, many genes related with ubiquitination are up- or downregulated in the absence of *AtVOZ1* and *AtVOZ2* in Arabidopsis (Kumar et al., 2018; Prasad et al., 2018). These results suggest that AtVOZ1 and AtVOZ2 modulate As tolerance, uptake, and translocation through the regulation of As uptake transporters or/and at a post-transcriptional level. Because AtVOZ1 and AtVOZ2 specifically recognize GCGTNx7ACGC, we looked for this sequence in the promoter region of *AtNIP1;1* using PatMatch (https://www.arabidopsis.org/cgi-bin/patmatch/nph-patmatch.pl). However, the 3-kb upstream sequences of *AtNIP1;1* do not contain the GCGTNx7ACGC element, suggesting that the gene is probably not a direct target of AtVOZ1 and AtVOZ2 but that the VOZ transcription factors might regulate a factor regulating *AtNIP1;1*.

AtVOZ1 and AtVOZ2 were specifically expressed in different tissues (**Figure 3**) and the patterns of As allocation mediated by VOZ1 and VOZ2 were different (**Figure 2A**). *atvoz2* increased As accumulation in rosette leaves and stems, whereas *atvoz1* did not exhibit altered As accumulation. However, in Arabidopsis *voz1* and *voz2* double-knockout plants, As accumulation was reduced in seeds, whereas more As was accumulated in seed valves, stems, and roots compared with the WT (**Figure 2B**). The drastically altered As accumulation in the double mutant might not be caused by delayed development shown in the *atvoz1 atvoz2* mutant. Because altered development time caused by different light periods did not change As accumulation in the WT (**Figures 2A, B**), and, furthermore, As translocation mainly is regulated by As transporters located at the plasma membrane of roots and vacuolar membrane in phloem cells (Isayenkov and Maathuis, 2008; Kamiya et al., 2009; Song et al., 2014; Xu et al., 2015; Lindsay and Maathuis, 2016). The results suggest that interaction of the different targets of AtVOZ1 and AtVOZ2 might have additive effects on As translocation into various tissues. It has been suggested that OsVOZ1 acts as a negative transcriptional regulator, whereas OsVOZ2 is a transcriptional repressor (Wang et al., 2020). Therefore, to elucidate the direct molecular functions of VOZ1 and VOZ2 under various conditions *in planta*, identification of the targets of each gene based on transcriptomic analysis using each single mutant and examination of the interactions between the target genes of VOZ1 and VOZ2 are required.

### 4.3 VOZs regulate As translocation into seeds

Many studies have indicated that phloem loading and unloading of As are critical determinants of As levels in seeds. Excised rice panicles subjected to stem girdling (to damage phloem cells) exhibited highly reduced As levels in grains (10% of the control) (Carey et al., 2010). The *osabcc1* mutant, with reduced As compartmentalization into phloem cells, accumulates 10-fold more As in grains than the WT (Song et al., 2014). *atnip7;1* knockout plants exhibit reduced As concentrations in phloem sap and seeds (Lindsay and Maathuis, 2016). Arabidopsis knockout mutants of the inositol transporters *AtINT2* and *AtINT4*, which are highly expressed in phloem, show reduced As loading in phloem and As accumulation in seeds (Duan et al., 2016). However, prior to this study, no transcription factors have been shown to regulate As loading and unloading into or from the phloem tissue. Our findings indicate that Arabidopsis and rice VOZs regulate As translocation through the phloem (**Figure 2**). In line with this observation, As concentrations of Arabidopsis phloem sap, As translocation from shoots to roots, and As uptake activity in suspension cultured cells were clearly lower in the *atvoz1 atvoz2* double-knockout mutant than in the WT (**Figures 2C, D, F**); as consequence, the reduced phloem transport activity of As might cause the lower As concentration in seeds and higher level of As in roots, stem, and seed valves than those of WT (**Figures 1F**, **2B**, **Supplementary Figure 12B**). Together, our findings suggest that VOZs might function in As tolerance and accumulation by regulating transporter genes involved in the As phloem loading.

The loss of function of VOZs in Arabidopsis and rice reduced As accumulation in seeds while increasing As in seed valves and husks, indicating that the genes might regulate As partitioning between the two tissues. To explore how Arabidopsis and rice VOZs differentially regulates As accumulation in the seeds versus seed valves or caryopsis versus the husk, we analyzed tissue-specific GUS expression in the siliques and grains of *ProVOZ1::GUS-VOZ1* and *ProVOZ2::GUS-VOZ2* and *OsVOZ1p::GUS* plants (**Figure 3D**, **Figure 4F**). The VOZs promoter::GUS plant showed that Arabidopsis *VOZ1* and *VOZ2* were highly expressed at the funiculus and septum connecting seeds, and rice VOZ1-GUS signals were primarily detected in the ovular vasculature, and layers of tube cells and cross-cells, which are important sites for the transfer of nutrients to the endosperm during seed development (Krishnan and Dayanandan, 2003; Carey et al., 2010). These results suggest that Arabidopsis and rice VOZs affect As partitioning in seeds and tissues surrounding seeds through the regulation of some genes. The *OsABCC1*, *AtINT2*, and *AtINT4* also affect As concentration in the seeds by reducing the As content, whereas the knockout mutants accumulated more As in their seeds compared with those of WTs (Song et al., 2014; Duan et al., 2016). However, *AtINT2* and *AtINT4* transcript levels in *atvoz1 atvoz2* were similar to those of the WT (**Figure 5A**), indicating that these transporters are regulated by other transcription factors. Because As import and export is catalyzed by transporters, our results suggest that aquaporins [plasma membrane intrinsic proteins (PIPs), NOD26-like intrinsic proteins (NIPs)] and hexose transporters localized to the plasma membrane could be either direct or indirect downstream targets of AtVOZ1 and AtVOZ2, because they are major transporters involved in As uptake in Arabidopsis and yeast (*S. cerevisiae*) (Liu et al., 2004; Ma et al., 2008; Duan et al., 2016; Li et al., 2016).

In this study, we demonstrated that the transcription factors VOZ1 and VOZ2 regulate As accumulation in seeds by modulating As transport into the phloem. To deliver specific substrates to particular tissues at the appropriate time, transcription factors must regulate the expression of transporters localized in the vascular bundle or proteins that modulate their activities. We showed that rice and Arabidopsis VOZs are expressed in phloem cells and thereby play critical roles in determining the As content of seeds. Therefore, this study lays the foundation for developing rice lines that exhibit a strongly decreased accumulation of As in the grain. However, it needs further studies to elucidate As transport specific downstream targets of VOZs without jeopardizing agronomic traits, to develop a rice with lower grain As, because rice and Arabidopsis VOZs exhibit various adverse physiological and developmental functions.

## Data availability statement

The RNA sequencing data presented in the study are deposited in the NCBI SRA repository, reference number PRJNA992992.

## Author contributions

YW: investigation, formal analysis, and writing—original draft preparation. CC: investigation, formal analysis, writing—original draft preparation, and review and editing. KV: investigation, formal analysis, and review and editing. JL, JZ, D-YK, TP, C-YZ, and M-HW: investigation and formal analysis. EM: review and editing. J-SJ: investigation, formal analysis, and review and editing. W-YS: conceptualization, investigation, formal analysis, writing—original draft preparation, review and editing, supervision, and funding acquisition. All authors contributed to the article and approved the submitted version.

## Supplementary material

Supplementary Table 3 RNA sequence analysis from node I samples of *osvoz1* and *osvoz2* and their isogenic WT.
