# Supplementary material for: VOZ1 and VOZ2 transcription factors regulate arsenic tolerance and distribution in rice and Arabidopsis
Source: Front Plant Sci. 2023 Sep 20;14:1209860. doi: 10.3389/fpls.2023.1209860 (PMC10548236; doi:10.3389/fpls.2023.1209860)
Supplement: Supplementary file 1 [file DataSheet_1.docx]

Supplementary Material

**VOZ1 and VOZ2 transcription factors regulate arsenic tolerance and distribution in rice and Arabidopsis**

**Ying Wen^1,^**^†^**, Chayanee Chairattanawat^2,^**^†^**, Kieu Thi Xuan Vo^3,^**^†^**, Jiayou Liu^1^, Jie Zhang^1^, Ting Pan^1^, Do-Young Kim^4^, Enrico Martinoia^5^,** **Chun-Yan Zhong^6^, Mao-Hui Wang^6^, Jong-Seong Jeon^3,*^, Won-Yong Song****^1,2^****^,^****^*^**

^1^ Department of Horticulture, Foshan University, Foshan, Guangdong, China

^2^ Department of Integrative Bioscience and Biotechnology, Pohang University of Science and Technology, Pohang, Republic of Korea.

^3^ Graduate School of Green-Bio Science and Crop Biotech Institute, Kyung Hee University, Yongin, Republic of Korea

^4^ Advanced Bio-convergence Center, Pohang Technopark, Pohang, Republic of Korea

^5^Institute of Plant Biology, University Zurich, Zollikerstrasse 107, Zurich, Switzerland

^6^Zhaoqing Institute of Agricultural Sciences, Zhaoqing 526070, China

†These authors contributed equally to this work

**^*^Correspondence:**

Jong-Seong Jeon

E-mail: [jjeon@khu.ac.kr](mailto:jjeon@khu.ac.kr)

Won-Yong Song

E-mail: [songwy@fosu.edu.cn](mailto:songwy@fosu.edu.cn)

**1 Supplementary Figures and Tables**

**1.1 Supplementary Figures**


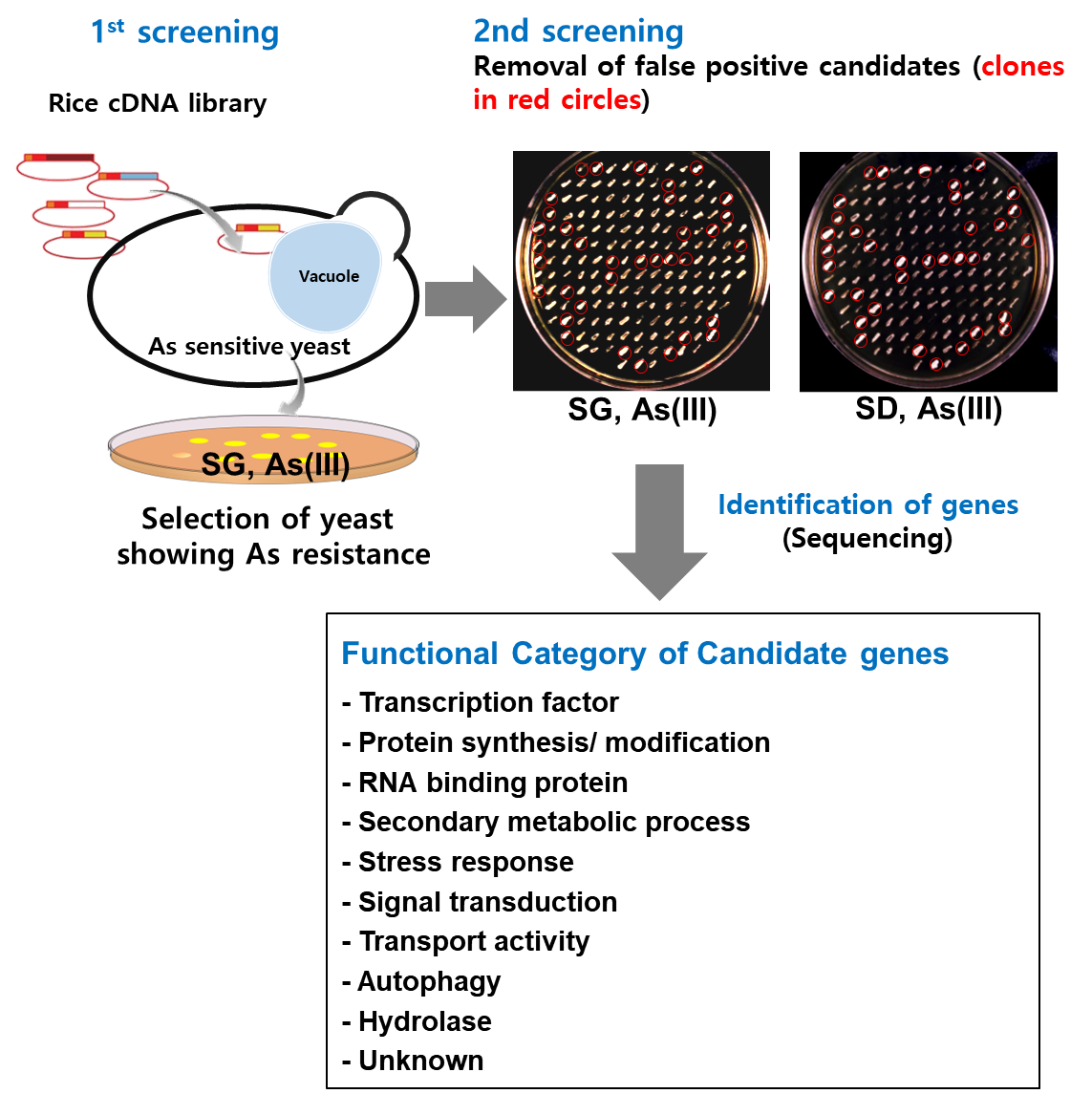


**Supplementary Figure 1. Screening for As tolerance genes expressed in rice roots using a yeast expression system.** A rice cDNA library was constructed using the pYES2 vector containing the GAL promoter. SM15 yeast cells transformed with a cDNA library generated from rice roots treated with As were grown on SG medium containing As(III) to select yeast clones exhibiting As tolerance. False-positive clones surviving on SD supplemented with As were eliminated because the rice genes were not expressed under these conditions in the absence of galactose. Plasmids were rescued from the candidate clones, and target genes were identified.


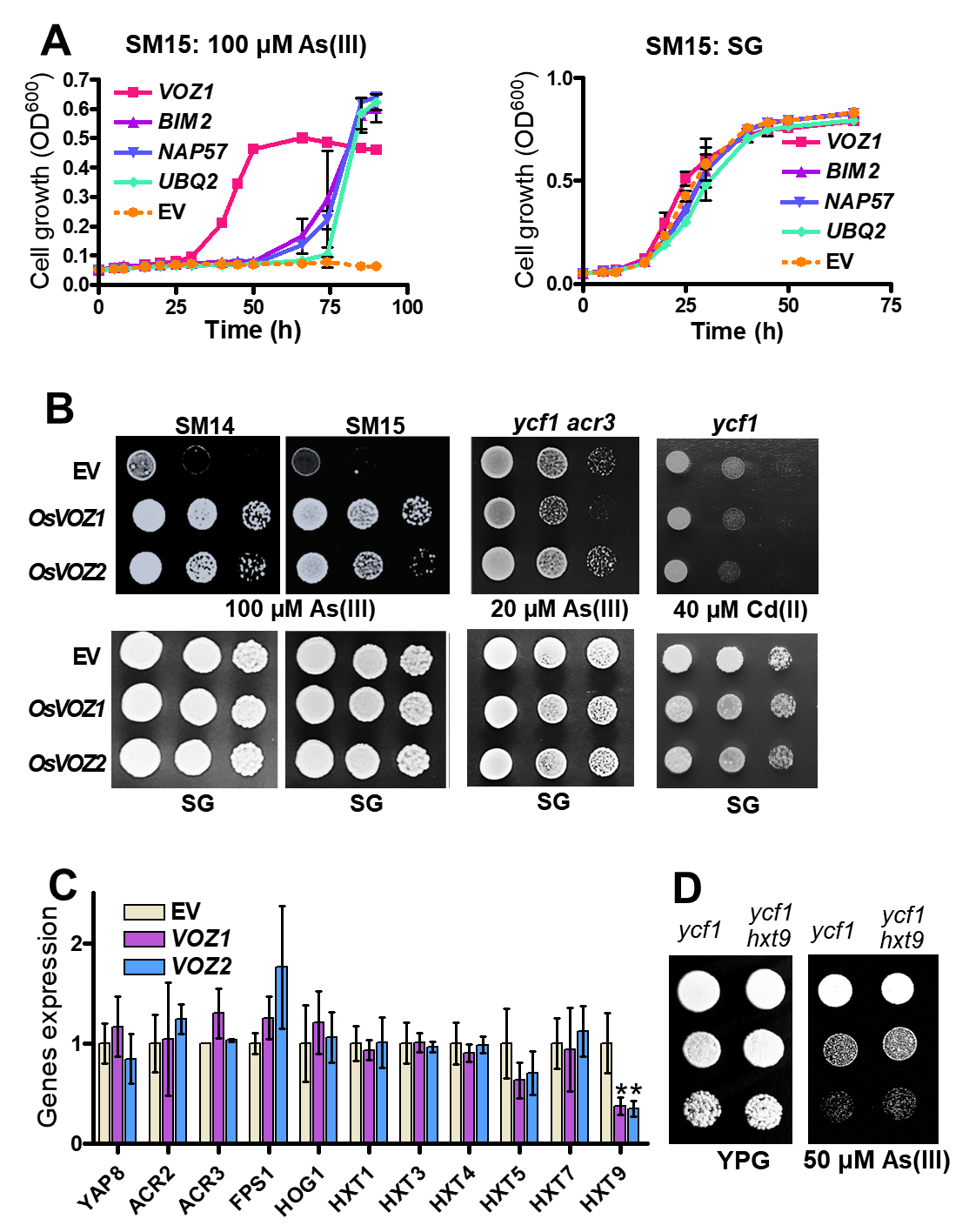


**Supplementary Figure 2.** **Functional analysis of OsVOZ1 and OsVOZ2 for As resistance in yeast.** A Comparison of candidate genes for As resistance in SM15 yeast cells grown in liquid medium supplemented with or without 100 μM As(III) (n=5). (B) The effects of OsVOZ1 and OsVOZ2 on As and Cd resistance in various yeast strains. (**C**) Transcript levels of yeast genes participating in As transport in SM14 yeast cells transformed with *OsVOZ1*, *OsVOZ2*, or empty vector. Yeast cells were treated with 100 µM As(III) for 3 h. Data are means ± SD (HXT9, n = 3; others, n = 2). Statistical significance was calculated using Student’s *t*-test (**P* < 0.05). (D) Comparison of As(III) sensitivity in *ycf1* and *ycf1 hxt7* yeast knockout mutants. SG: synthetic galactose medium, YPG: yeast extract, peptone and galactose medium


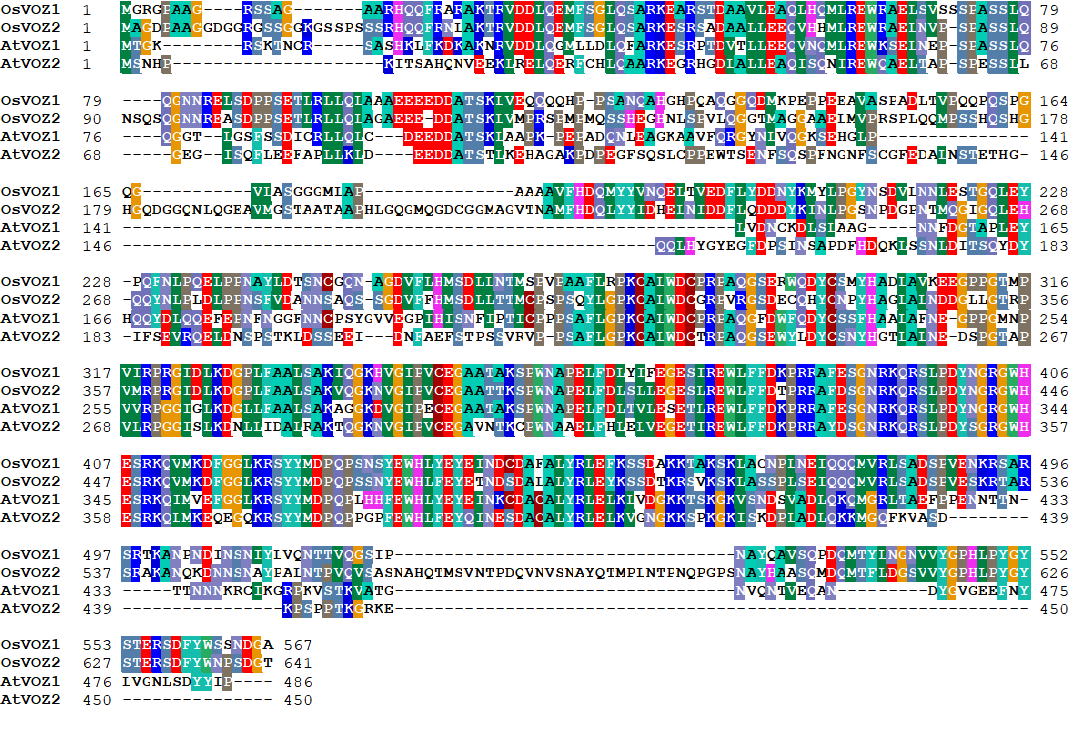


**Supplementary Figure 3.** Comparison of amino acid sequences of VOZ1 and VOZ2 from rice and Arabidopsis. Highlighted columns represent amino acids conserved in these proteins. The data was developed using BioEdit sequence alignment program.

**
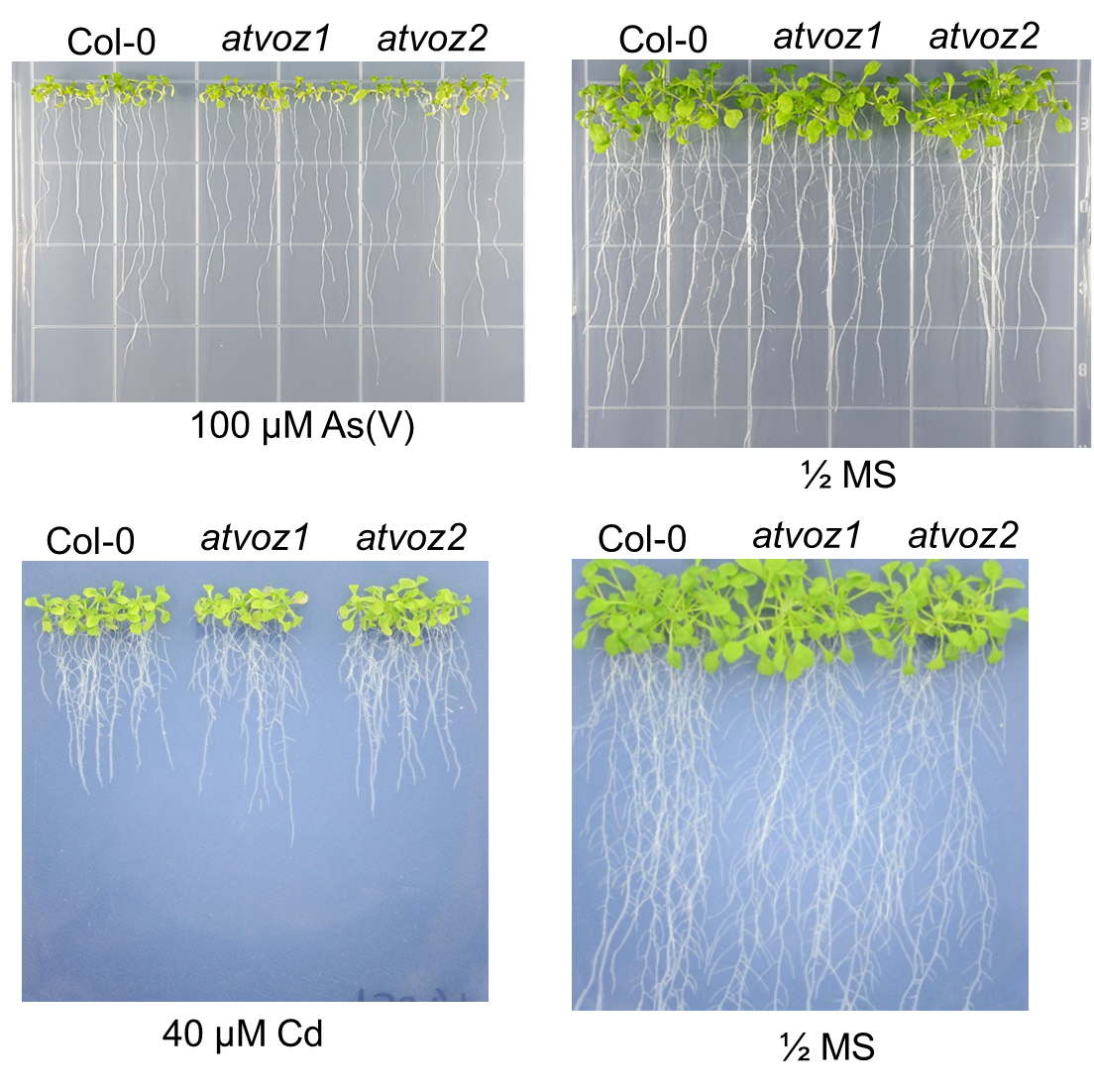
**

**Supplementary Figure 4. As and Cd sensitivity of *atvoz1* and *atvoz2* single mutants.** Phenotypic comparison of *atvoz1* and *atvoz2* knockout mutants and the corresponding WT on 1/2 MS agar plates supplemented with or without As (V) for 4 weeks.

**
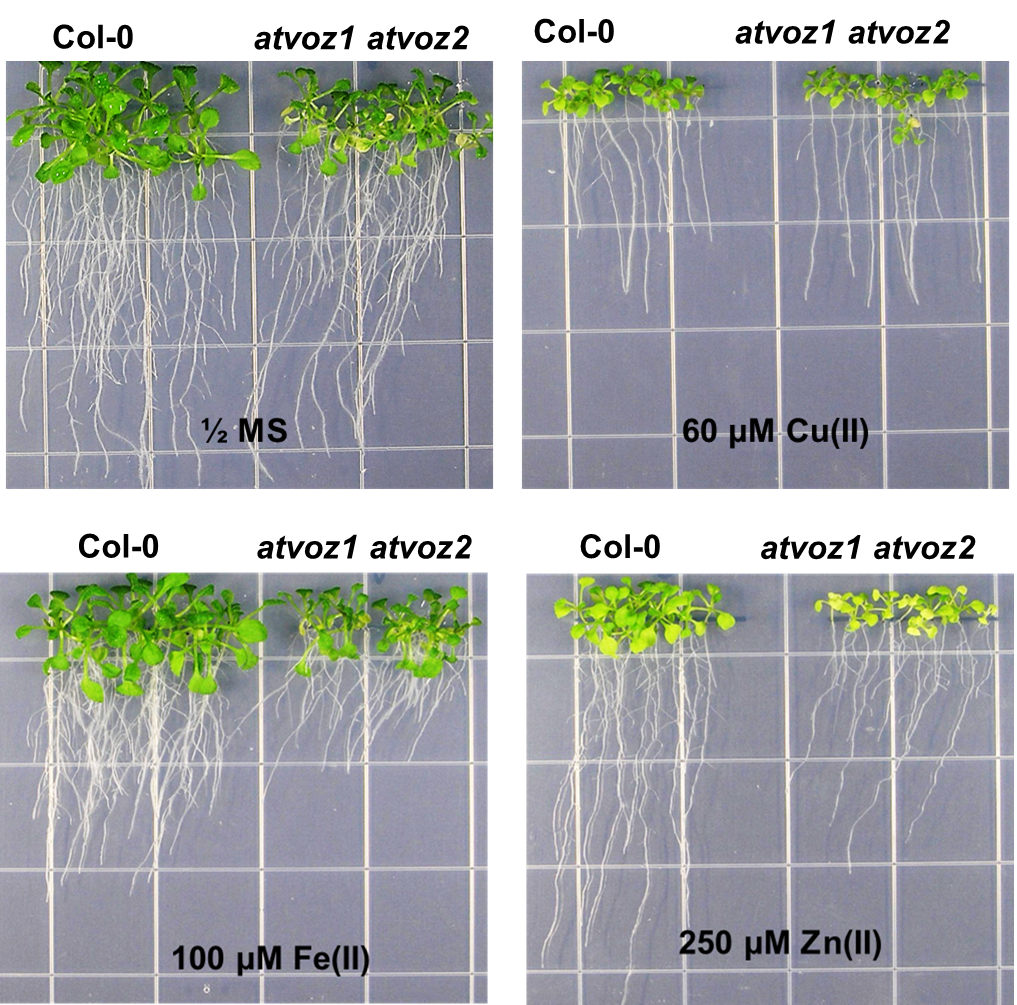
**

**Supplementary Figure 5. Comparison of metal sensitivity in *atvoz1atvoz2* and wild type plants.** The *atvoz1 atvoz2* knockout and WT were cultured on 1/2 MS agar plates supplemented with or without CuSO_4_, FeSO_4_ and ZnCl_2_ for 4 weeks.

**Supplementary Figure 6. Comparison of As efflux activity in roots of WT and *atvoz1 atvoz2* mutant.** Plants were grown in hydroponic medium for 5 weeks then supplemented with 10 µM As(V) for 12 hrs, transferred into new hydroponic medium without As, incubated for the indicated time, and analyzed As efflux. Data was calculated from 5 replicates as the mean (±SE).


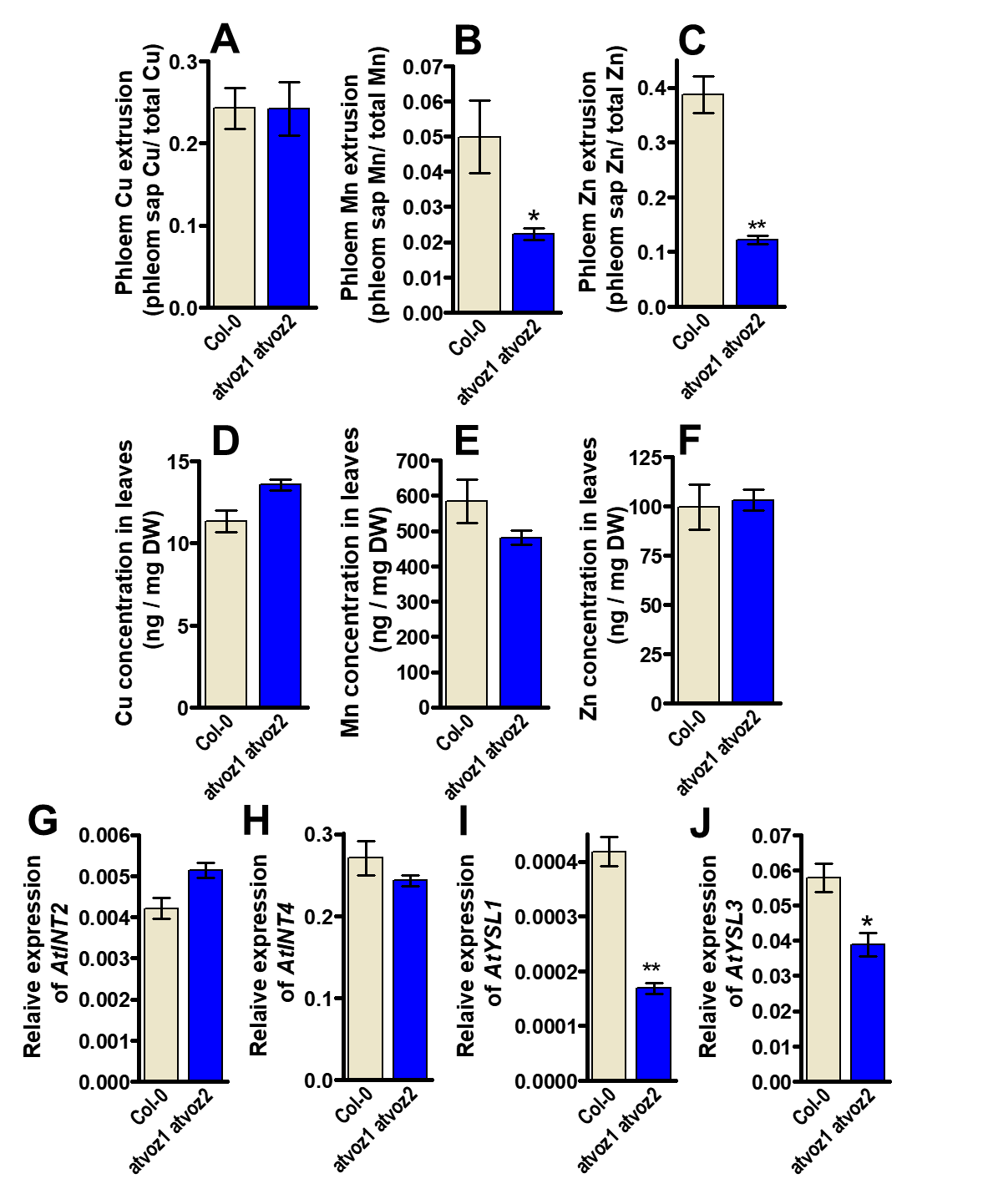


**Supplementary Figure 7. Metal (loid)s concentrations in phloem exudates and leaves, and expression of phloem transporter genes of metal (loid)s in *atvoz1 atvoz2* and wild-type plants.** (**A-C**) Concentrations of Cu, Mn and Zn in phloem exudates. Eight-week-old Arabidopsis plants cultured in hydroponic medium were treated with 5 µM As(III) for 3 days, and phloem exudates were collected using the K_2_-EDTA method. (**D-F**) Concentrations of As and metals in leaves from eight-week-old hydroponic cultured Arabidopsis plants treated with 5 µM As(III) for 3 days, Bars indicate means ±SE (n=6 plants). (G-J) The expression level of *AtINT2*, *AtINT4*, *AtYSL1* and *AtYSL3*  in Col-0 and *atvoz1 atvoz2*. The values were normalized by *Actin2* values (n=6). The statistical significance was calculated using Student’s *t* test (**P*<0.05, ***P*<0.001).


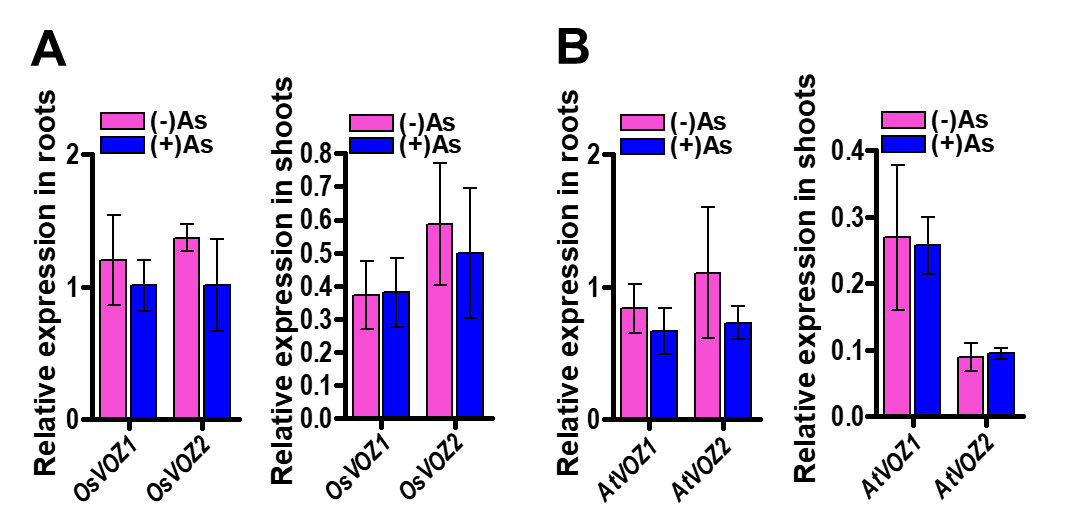


**Supplementary Figure 8. Expression levels of rice (A) and Arabidopsis (B) VOZ1 and VOZ2 in roots and shoots in response to As treatment.** Three-week old rice or four-week old Arabidopsis plants were treated with 10 μM As(III) or 100 μM As(V) for 5 h. Bars indicate means ± SD (n = 3).


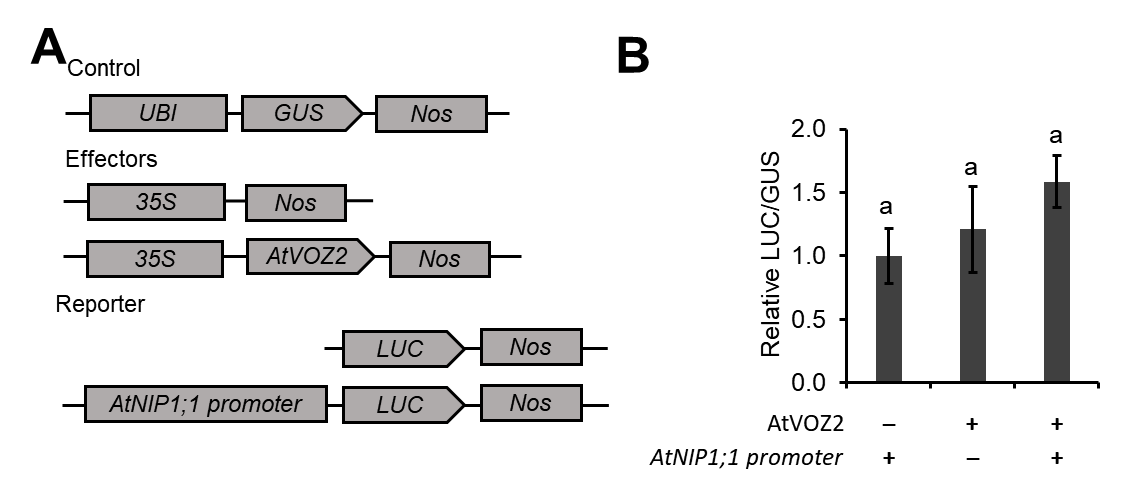


**Supplementary Figure 9. AtVOZ2 did not activate *AtNIP1;1* expression.** (A) Schematic diagrams of the constructs used. *GUS*, *β-glucuronidase*; *Nos*, nopaline synthase terminator; *LUC*, firefly luciferase gene; *UBI*, maize *Ubiquitin1* promoter; *35S*, CaMV 35S promoter. (B) Relative LUC/GUS activity in transfected protoplasts. Different letters indicate significantly different means (Tukey's multiple comparison analysis, *P* ≤ 0.05).


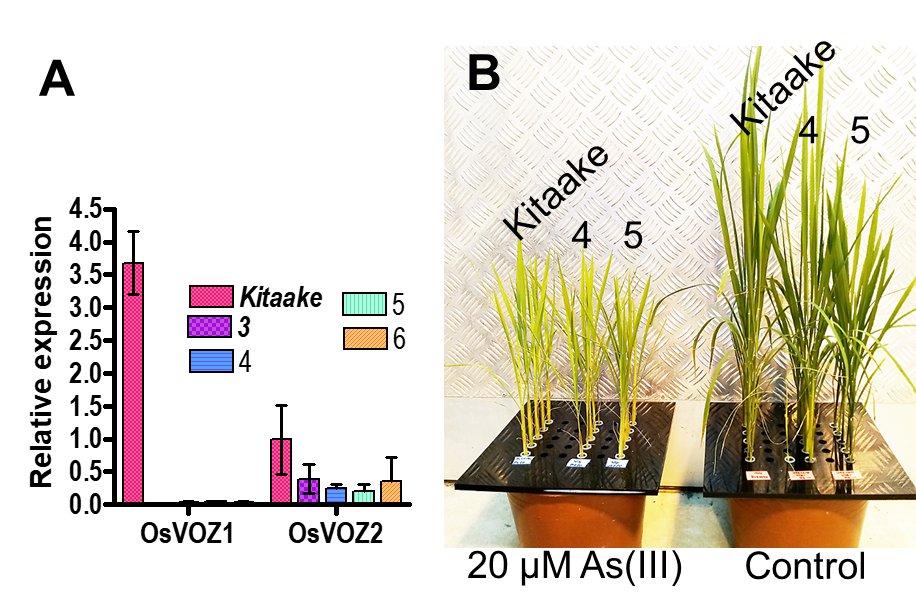


**Supplementary Figure 10. Phenotypic analysis of loss-of-function *voz1* *voz2* rice mutants.** (**A**) qRT-PCR of *OsVOZ1* and *OsVOZ2* expression in UBI1300::OsVOZ1 transgenic and WT plants (Kitaake). The expression level of each gene was normalized to that of *OsActin*. Bars indicate means ± SD (n = 3). (**B**) Phenotypic comparison of T2 UBI1300::OsVOZ1 transgenic and WT plants (Kitaake) in hydroponic medium with or without 20 μM As(III).


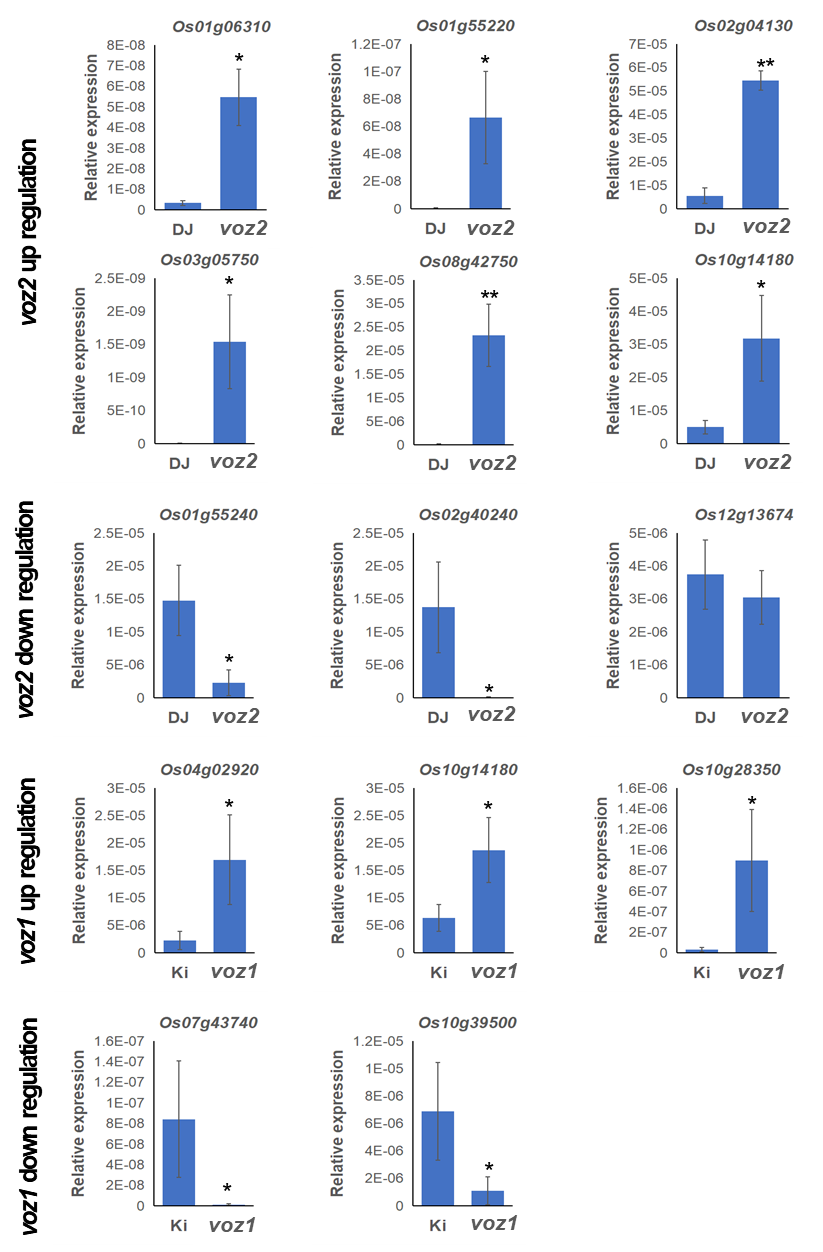


**Supplementary Figure 11. Confirmation of RNA-seq result using quantitative RT-PCR.** Quantitative RT-PCR was performed using RNA from rice nodal tissues. The values were normalized using Actin2 values (n=3). Bars indicate means ± SE. The statistical significance was calculated using Student’s *t* test, (**P*<0.05, ***P*<0.001).


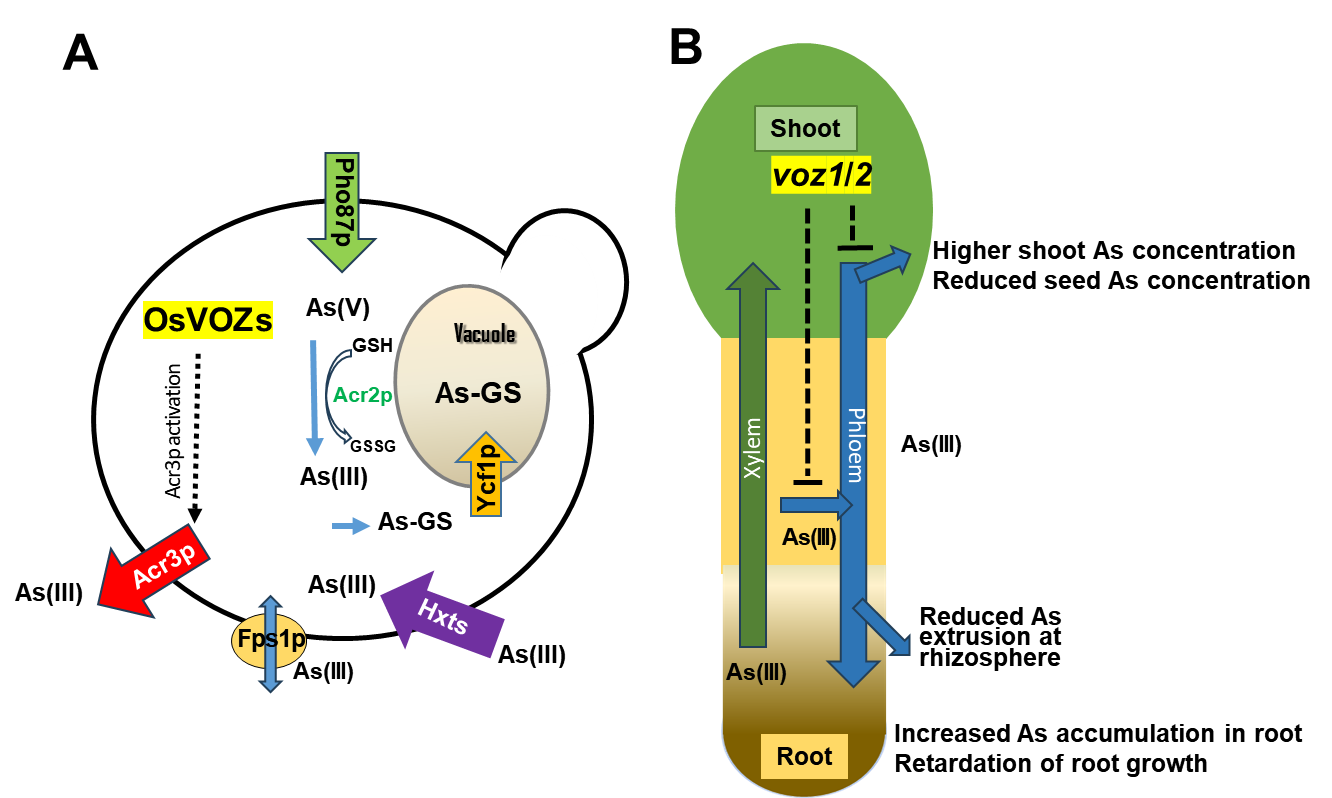


**Supplementary Figure 12. The role of VOZ1/2 for As tolerance and transport in yeast and plants.** (**A**) OsVOZ1 and OsVOZ2  enhance the protein amount of As(III) efflux transporter Aar3p. As a result, the yeast cells reduce cytosolic As concentration and increase As tolerance. (**B**) The *atvoz1 atvoz2* double knockout mutant exhibits reduced As phloem transport activity and reduced As extrusion at rhizosphere. As a result, the mutant accumulates more As in roots and shoots, decreases As tolerance and exhibits lower seeds As concentration compared to WT.

**1.2 Supplementary Tables**

**Table S1.** Novel arsenic resistance genes from rice that enhance arsenic tolerance in a yeast mutant.

| Functional Category | Locus ID (RAP) | MSU | Description |
| --- | --- | --- | --- |
| Transcription Factor | Os01g0753000 | LOC_Os01g54930 | Transcription factor VOZ1 |
|  | Os02g0181300 | LOC_Os02g08440 | WRKY transcription factor 71 |
|  | Os09g0475400 | LOC_Os09g29930 | Transcription factor BIM2 |
| Proteolysis | Os02g0161900 | LOC_Os02g06640 | Polyubiquitin RUBQ2 |
|  | Os01g0316800 | LOC_Os01g21440 | probable 26S proteasome complex subunit sem1 |
|  | Os05g0588900 | LOC_Os05g51130 | AAA-ATPase |
|  | Os07g0512200 | LOC_Os07g32800 | Autophagy-related protein 8A |
| RNA Binding Protein | Os07g0636000 | LOC_Os07g44190 | H/ACA ribonucleoprotein complex subunit 4; NAP57 |
|  | Os12g0632000 | LOC_Os12g43600.1 | glycine-rich RNA-binding protein AtGRP7 |
|  | Os04g0346100 | LOC_Os04g27860 | 40S ribosomal protein S27 AT3G61110 |
| Secondary Metabolic Process | Os03g0278200 | LOC_Os03g17000 | NAD-dependent epimerase/dehydratase family protein. |
|  | Os10g0527400 | LOC_Os10g38340 | Similar to Tau class GST protein 3. |
| Stress Response | Os03g0299600 | LOC_Os03g18770 | Wound-induced WI12 family protein. |
|  | Os03g0353400 | LOC_Os03g23010 | Similar to Early response to dehydration 15-like protein |
| Signal Transduction | Os07g0195100 | LOC_Os07g09680 | Similar to Ras-related protein ARA-3. |
| Transport Activity | Os05g0430900 | LOC_Os05g35594 | Peptide transporter, |
|  | Os07g0557100 | LOC_Os07g37100 | Equilibrative nucleoside transporter family protein. |
|  | Os08g0478700 | LOC_Os08g37370 | Mitochondrial carrier protein |
| Hydrolase | Os06g0354700 | LOC_Os06g24730 | Hydrolase, alpha/beta fold family domain containing protein |
| Unknown | Os01g0231800 | LOC_Os01g13090 | Nucleic acid binding protein |
|  | Os01g0793900 | LOC_Os01g58140.1 | Conserved hypothetical protein. |
|  | Os03g0162900 | LOC_Os03g06710.1 | Pentatricopeptide repeat domain containing protein |
|  | Os03g0314200 | LOC_Os03g19970 | CAS1 domain-containing protein 1 precursor |
|  | Os04g0496600 | LOC_Os04g41920 | Protein FAM133, Similar to H0306B06.6 protein. (Os04t0496600-01) |
|  | Os05g0311500 | LOC_Os05g24650 | Protein of unknown function DUF567 family protein. |
|  | Os08g0331800 | LOC_Os08g24300 | Conserved hypothetical protein. |

**Table S2.** Yeast strains and primers used in this study.

| Strain/ Primer | Genotype/Sequence | Purpose | Ref. |
| --- | --- | --- | --- |
| DTY167 | *MATα ura3-52 his6 leu2-3,112 his3-Δ,200 trp1-901 lys2-80 suc*2̄ *Δycf1∷hisG* | As phenotype analysis | Szczypka et al. 1994 |
| RW105 | W303-1A *acr3D::kanMX ycf1Δ::loxP* | As phenotype analysis | Maciaszczyk-Dziubinska et al. 2010 |
| SM14 | *MATα, ura3, leu2, his3, trp3, lys2, suc2, ycf::hisG, yhl035c::Leu3, yll015w::Kan-MX6, yll048c:: TRP1-MX6* | As phenotype analysis | Mendoza-Cozatl et al. 2010 |
| SM15 | SM14, *TaPCS1::cup1-1* | As phenotype analysis | Mendoza-Cozatl et al. 2010 |
| yll039cF | AGAATTCAGTGTCATTTGAGGGC | yll039c::Kan-MX6 amplification | This study |
| yll039cR | ATGATAGGACACTATAAAGGTGG | yll039c::Kan-MX6 amplification | This study |
| CYC1 | AGGGCGTGAATGTAAGCGTGAC | pYES2 vector sequence | This study |
| T7 | TAATACGACTCACTATAGGG | pYES2 vector sequence | This study |
| AtVOZ2aF | ATGTCAAACCACCCGAAGAT | genotyping/ gene expression | Yasui et al. 2012 |
| AtVOZ1RT-F | GCAACTGCTAAATCTCCATGGAATGCTCCAG | qRT-PCR | This study |
| AtVOZ1RT-R2 | CATGTTCTGACTTCCCCTGAACC | qRT-PCR | This study |
| AtVOZ2RT-F | GTTAACACAAAATGCCCATGGAACGCAGCAG | qRT-PCR | This study |
| AtVOZ2RT-R | TCACTCCTTACGACCTTTGGT | qRT-PCR | This study |
| AtTUB8-F | CTCACAGTCCCGGAGCTGACAC | qRT-PCR | This study |
| AtTUB8-R | GCTTCAGTGAACTCCATCTCGT | qRT-PCR | This study |
| OsVOZ1RT-F | TCAGAACACTACAGTTCAAGGCAG | qRT-PCR | This study |
| OsVOZ1RT-R | AGGTTACAAGTCTAGCAAACCTGG | qRT-PCR | This study |
| OsVOZ2-RT-F | GCCGATGCAATCTTCTCATGAGGG | qRT-PCR | This study |
| OsVOZ2-RT-R | ATTGGGGCCGTCTGGGTTTGAGC | qRT-PCR | This study |
| OsActinRT-F | GACTCTGGTGATGGTGTCAGC | qRT-PCR | Song et al. 2014 |
| OsActinRT-R | GGCTGGAAGAGGACCTCAGG | qRT-PCR | Song et al. 2014 |
| ScALG9RT-F | CACGGATAGTGGCTTTGGTGAACAATTAC | qRT-PCR | Teste, Marie-Ange et al. 2009 |
| ScALG9RT-R | TATGATTATCTGGCAGCAGGAAAGAACTTGGG | qRT-PCR | Teste, Marie-Ange et al. 2009 |
| HXT1RT-F | ATGAAGCACCACGACGGTAG | qRT-PCR | This study |
| HXT1RT-R | ACCACCGACACCTAAACCAG | qRT-PCR | This study |
| HXT3RT-F | AGCTAGAGCTGCTGGTTCAG | qRT-PCR | This study |
| HXT3RT-R | ATTGGAGCCCAAGTGGTAGC | qRT-PCR | This study |
| HXT4RT-F | ACATTGGTTGTGCCATTGGTGG | qRT-PCR | This study |
| HXT4RT-R | GCCCAAGCGAAACCTAGACC | qRT-PCR | This study |
| HXT5RT-F | CCCGTGCAAACGGGACTACC | qRT-PCR | This study |
| HXT5RT-R | ACCAGAGTACCACGCAACTG | qRT-PCR | This study |
| HXT7RT-F | GCTGCATCCATGACTGCTTG | qRT-PCR | This study |
| HXT7RT-R | CGTCGTAGTTGGCACCTCTTC | qRT-PCR | This study |
| HXT9RT-F | CTGCTTTGCTGTGTTTGCCTC | qRT-PCR | This study |
| HXT9RT-R | TGGTCTATGGCGTCAGCATC | qRT-PCR | This study |
| ACR2RT-F | AGGCAACTCAAGGCCTAAT | qRT-PCR | Amaral C et al. 2013 |
| ACR2RT-R | GAACATGCCAAGCGTTTGTA | qRT-PCR | Amaral C et al. 2013 |
| ACR3RT-F | CGGCATACCACTGGGAATT | qRT-PCR | Maciaszczyk-Dziubinska et al. 2010 |
| ACR3RT-R1 | GCACCAATGGGACAAAGCA | qRT-PCR | Maciaszczyk-Dziubinska et al. 2010 |
| Yap8RT-f | GAGGAACATGCCTTCTGATGAACGG | qRT-PCR | Amaral C et al. 2013 |
| Yap8RT-R | CAACGGTGACGGCACTGTACAATTC | qRT-PCR | Amaral C et al. 2013 |
| Fps1RT-F | CCATCCCGGAGTCACATTTATCGAG | qRT-PCR | This study |
| fps1RT-R | TTTCTGCAGAAGACCCGGTAACG | qRT-PCR | This study |
| HOG1RT-F2 | GACATTTGGTCCGCTGGTTG | qRT-PCR | This study |
| HOG1RT-R2 | TACGGCATCAGGTTCGACTG | qRT-PCR | This study |
| AtNIP1;1-RT-F | ATTGCCTTCGCCTCTTGTGGCCGTTTC | qRT-PCR | Xu W. et al. 2015 |
| AtNIP1;1-RT-R | ACCCATGCACCCGCAATCGCACCAAG | qRT-PCR | Xu W. et al. 2015 |
| AtNIP1;2-RT-F | ATAGAGCGATCGGAGAACTTGCTGG | qRT-PCR | This study |
| AtNIP1;2-RT-R | GCACGTAATTAGATATGGCCAAACTCG | qRT-PCR | This study |
| AtNIP3;1-RT-F | TTGATTGGAGAGTTCGTGGGGACAT | qRT-PCR | Xu W. et al. 2015 |
| AtNIP3;1-RT-R | CCGGAGAAAAGGATGTCGAGTACTATG | qRT-PCR | Xu W. et al. 2015 |
| AtNIP5;1-RT-F | CACCGATTTTCCCTCTCCTGATGTC | qRT-PCR | Tanaka et al. 2011 |
| AtNIP5;1-RT-R | CGCATGCAGCGTTACCGATTAGG | qRT-PCR | Tanaka et al. 2011 |
| AtNIP6;1-RT-F | CTCAATCCGGCTGTAACCATTGCC | qRT-PCR | This study |
| AtNIP6;1-RT-R | TTGGGTGCTTCATCTTCCTCTGGC | qRT-PCR | This study |
| AtNIP7;1-RT-F | GGCCACGTGGGATTGCTAGAGTAC | qRT-PCR | This study |
| AtNIP7;1-RT-R | GATGAGGACCACAATGCAACGCGG | qRT-PCR | This study |
| AtINT2-RT-F | GCTTTCTCTGCCGGAATCGGTGG | qRT-PCR | This study |
| AtINT2-RT-R | CACGTTCCGGTCACATCAGTAAAGG | qRT-PCR | This study |
| AtINT4RT-F | CTCCGGCGAGGATTAGAGGTGC | qRT-PCR | This study |
| AtINT4RT-R | TGATACCAGCAGCGAGTCCACG | qRT-PCR | This study |
| AtCLT3RT-F | GTGATCGACGGAGAGCATGTTAAG | qRT-PCR | This study |
| AtCLT3RT-R | GAGCAGCACCTGATCCACTTG | qRT-PCR | This study |
| AtVOZ2_TOPO_F | CACCATGTCAAACCACCCGAAGATCAC | 35Spro::AtVOZ2 | This study |
| AtVOZ2_TOPO_R | TCACTCCTTACGACCTTTGGTTG | 35Spro::AtVOZ2 | This study |
| AtNIP1;1_pro_TOPO_F | CACCCCATGGCACCTTTTAAATCAAACT | AtNIP1;1::LUC | This study |
| AtNIP1;1_pro_TOPO_R | TTGGCAAACATGGGGGAGAG | AtNIP1;1::LUC | This study |
| AtYSL1-F | TCCCAATG-TGGTTCGCAGTTT | qRT-PCR | This study |
| AtYSL1-R | TTGAGACCGCAGCGAATGTA | qRT-PCR | This study |
| AtYSL3-F | GTGGCGGCAAATCTCGTTA | qRT-PCR | This study |
| AtYSL3-R | CCATCGGTAATGGAACCCAAT | qRT-PCR | This study |
| At18s-F | CGGCTACCACATCCAAGGAA | qRT-PCR | This study |
| At18s-R | GCTGGAATTACCGCGGCT | qRT-PCR | This study |
| Os10g39500-F | AGAGATTGCTGTCAGTCGGC | qRT-PCR | This study |
| Os10g39500-R | GGATGTCCTTTCTGGTCGCA | qRT-PCR | This study |
| Os07g43740-F | ACCGGTTCCACAGCGAGT | qRT-PCR | This study |
| Os07g43740-R | CCTAATGTTCCATGGCTCCCC | qRT-PCR | This study |
| Os04g02920-F | GGCATTCGAGGGGAATGACA | qRT-PCR | This study |
| Os04g02920-R | AGGCACAAATTCCCAACCCA | qRT-PCR | This study |
| Os10g28350-F | CCAGAGGCTTCCTCATCACC | qRT-PCR | This study |
| Os10g28350-R | TCCCTGCAGGCAGAACAATC | qRT-PCR | This study |
| Os10g14180-F | TGCTCTCGAAGGTCTGCATC | qRT-PCR | This study |
| Os10g14180-R | AAGTACGTGTGCTCGTCAGG | qRT-PCR | This study |
| Os08g42750-F | GCCATCGACTTCAACAGGGA | qRT-PCR | This study |
| Os08g42750-R | TCTGCACGTACTTGTCCACC | qRT-PCR | This study |
| Os01g55220-F | CGGCGTCGACCAAGGC | qRT-PCR | This study |
| Os01g55220-R | TCAATGCGCGAGGAAGGC | qRT-PCR | This study |
| Os10g14180-F | TGCTCTCGAAGGTCTGCATC | qRT-PCR | This study |
| Os10g14180-R | AAGTACGTGTGCTCGTCAGG | qRT-PCR | This study |
| Os03g05750-F | GGACAAAGGCAAGAGCATCG | qRT-PCR | This study |
| Os03g05750-R | GTAGGTAGGGACGTCGTTGG | qRT-PCR | This study |
| Os01g06310-F | CATGACATGCACGGCGG | qRT-PCR | This study |
| Os01g06310-R | CATGTGCCCTCCATGTGGC | qRT-PCR | This study |
| Os02g04130-F | AGGACAAGTTCGCCTTCCTG | qRT-PCR | This study |
| Os02g04130-R | GTCAGGTACGAGGCTTGCTG | qRT-PCR | This study |
| Os02g40240-F | TGACCTTGAGATGCGAGGTG | qRT-PCR | This study |
| Os02g40240-R | TAAACCCAAACGTCCGGGAG | qRT-PCR | This study |
| Os01g55240-F | TTCCGCGTGAACCACTACC | qRT-PCR | This study |
| Os01g55240-R | GTGGCAATGGTGCAATCCTC | qRT-PCR | This study |
| Os12g13674-F | ATGAAGGTGGCGGCGG | qRT-PCR | This study |
| Os12g13674-R | CGCAGTCACCACCAACCG | qRT-PCR | This study |
